# Mutual reinforcement between telomere capping and canonical Wnt signalling in the intestinal stem cell niche

**DOI:** 10.1038/ncomms14766

**Published:** 2017-03-17

**Authors:** Ting-Lin B. Yang, Qijun Chen, Jennifer T. Deng, Geetha Jagannathan, John W. Tobias, David C. Schultz, Shan Wang, Christopher J. Lengner, Anil K. Rustgi, John P. Lynch, F. Brad Johnson

**Affiliations:** 1 Department of Pathology and Laboratory Medicine, Perelman School of Medicine, University of Pennsylvania, Philadelphia, Pennsylvania 19104, USA; 2 Cell and Molecular Biology Program, Biomedical Graduate Studies, University of Pennsylvania, Philadelphia, Pennsylvania 19104, USA; 3 Penn Molecular Profiling Center, Perelman School of Medicine, University of Pennsylvania, Philadelphia, Pennsylvania 19104, USA; 4 Wistar Institute, University of Pennsylvania, Philadelphia, Pennsylvania 19104, USA; 5 Department of Animal Biology, School of Veterinary Medicine, University of Pennsylvania, Philadelphia, Pennsylvania 19104, USA; 6 Division of Gastroenterology, Department of Medicine, Abramson Cancer Center, Perelman School of Medicine, University of Pennsylvania, Philadelphia, Pennsylvania 19104, USA; 7 Institute on Aging, University of Pennsylvania, Philadelphia, Pennsylvania 19104, USA

## Abstract

Critical telomere shortening (for example, secondary to partial telomerase deficiency in the rare disease dyskeratosis congenita) causes tissue pathology, but underlying mechanisms are not fully understood. Mice lacking telomerase (for example, *mTR*^*−/−*^ telomerase RNA template mutants) provide a model for investigating pathogenesis. In such mice, after several generations of telomerase deficiency telomeres shorten to the point of uncapping, causing defects most pronounced in high-turnover tissues including intestinal epithelium. Here we show that late-generation *mTR*^*−/−*^ mutants experience marked downregulation of Wnt pathway genes in intestinal crypt epithelia, including crypt base columnar stem cells and Paneth cells, and in underlying stroma. The importance of these changes was revealed by rescue of crypt apoptosis and Wnt pathway gene expression upon treatment with Wnt pathway agonists. Rescue was associated with reduced telomere-dysfunction-induced foci and anaphase bridges, indicating improved telomere capping. Thus a mutually reinforcing feedback loop exists between telomere capping and Wnt signalling, and telomere capping can be impacted by extracellular cues in a fashion independent of telomerase.

## Abstract (web summary)

Mice lacking telomerase provide a model to study pathogenesis caused by critical telomere shortening. Here, the authors provide evidence that telomere shortening causes downregulation of Wnt signalling in intestinal crypts and that defects can be partially rescued by treatment with Wnt agonists.

Fundamental to intestinal epithelial homeostasis are intestinal stem cells (ISCs), including crypt base columnar (CBC) stem cells and quiescent ‘+4' ISCs. CBCs divide every few days and are positioned at the crypt base between Paneth cells, which together with underlying stroma, contribute to the CBC niche by providing intercellular Wnt signals^1^. Canonical Wnt proteins mediate short-range intercellular communication leading within target cells to the inhibition of glycogen synthase kinase 3 enzymes and thus the transcriptional activation functions of the T-cell factor/lymphoid enhancement factor (TCF/LEF) transcription factors^2^. In contrast to CBCs, quiescent ISCs divide infrequently, are located above the Paneth cell and CBC niche, and are Wnt independent^3^,^4^,^5^,^6^. Both ISC types can generate all intestinal epithelial cell types, a function apparently provided primarily by CBCs, with quiescent ISCs dividing to generate new CBCs on the occasion of their loss^5^,^6^. Frequent cell divisions by CBCs put them at risk for replication-dependent telomere shortening. To guard against such shortening, CBCs express high levels of telomerase, which at its core comprises a polymerase (TERT) and associated RNA template (TR)^2^,^7^.

People with genetic mutations causing partial deficiencies in telomerase activity typically display a spectrum of pathologies collectively termed dyskeratosis congenita (DC), which includes defects in highly proliferative tissues, including bone marrow and the epithelia of the skin and gastrointestinal tract, and in tissues that proliferate in response to injury, including liver and lung^8^. The premature loss of telomeres in DC can be modelled in mice by genetic inactivation of telomerase, for example, *mTert*^*−/−*^ or *mTR*^*−/−*^ mutants. Lab strains of mice have long telomeres, and thus initial generations lacking telomerase (for example, G1 or G2 *mTR*^*−/−*^mutants) are relatively unaffected by telomere shortening^9^,^10^. However, critical telomere shortening in late-generation mice yields pathology primarily in proliferative tissues such as intestine, characterized by elevated levels of apoptosis in the crypt epithelium^10^,^11^,^12^. Recent reports indicate reduced expression of Wnt pathway genes in the intestinal epithelium of such mice^13^,^14^, but the full extent and functional importance of these Wnt pathway deficits have not been explored.

Here we demonstrate broad downregulation of canonical Wnt pathway gene expression in the intestinal crypt epithelium and underlying stroma of late-generation *mTR*^*−/−*^ mice. These changes appear to reflect a regulatory response to critical telomere shortening, and involve upregulation of the miR34a microRNA (miRNA). We further show that Wnt pathway agonists rescue intestinal pathology, gene expression, and most remarkably, telomere capping itself, in a fashion that does not involve telomere lengthening. These findings reveal a positive feedback loop between telomere capping and Wnt pathway activity, and are distinct from previous suggestions of connections between Wnt and the catalytic subunit of telomerase, TERT (refs 15, 16, 17). They also raise the possibility of novel therapeutic approaches to diseases with underlying telomere dysfunction.

## Results

### Wnt gene expression declines in late-generation *mTR* ^*−/−*^ mice

**Figure 1:**
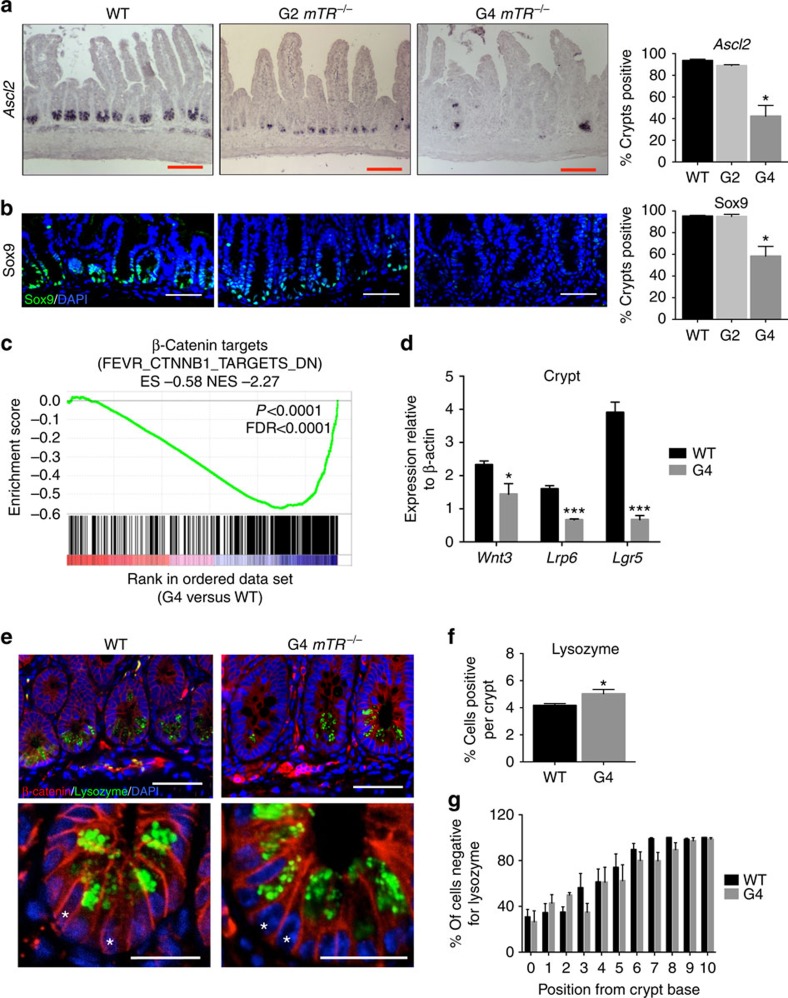
Defects in small intestinal CBC stem cell and Paneth cell gene expression in late-generation *mTR*^*−/−*^ mice. (**a**) Representative images of *in situ* hybridization for *Ascl2* transcripts in ileum from WT, G2 and G4 *mTR*^*−/−*^ mice and quantitation of crypts positive for staining (right; *n*=3); *P<0.05. Scale bars, 100 μm. (**b**) Representative images of Sox9 immunostaining in ileum from WT (*n*=5), 2nd generation (G2, *n*=3) and 4th generation (G4, *n*=4) *mTR*^*−/−*^ mice and quantitation of crypts positive for staining (right); *P<0.05. Scale bars, 50 μm. (**c**) Gene Set Enrichment Analysis (GSEA) revealed G4 *mTR*^*−/−*^ ileal crypts to have significantly reduced expression of genes downregulated in β-catenin knockout mouse crypts (FEVR_CTNNB1_TARGETS_DN) (*n*=3). Enrichment score (ES), normalized enrichment score (NES), *P* value and false discovery rate (FDR) are as described^50^ (see ‘Methods' section). (**d**) Quantitative reverse transcription PCR (qRT-PCR) analyses of gene expression for the Wnt ligand (*Wnt3*), Wnt co-receptor (*Lrp6*) and Wnt target gene and co-receptor (*Lgr5*) in WT and G4 *mTR*^*−/−*^ crypts (*n*=5). Also, note that Msi1 protein expression is unchanged (Supplementary Fig. 2d). *P<0.05 and ***P<0.005. (**e**) Immunostaining for Paneth cell lysozyme in WT and G4 *mTR*^*−/−*^ ileum. Enlarged insets show examples of cells at the normal CBC location between lysozyme-positive Paneth cells (asterisks). β-catenin staining marks cell peripheries. Scale bars, 50 μm (top), 25 μm (bottom). (**f**) Quantitation of Paneth cells as measured by lysozyme staining and (**g**) of cells intercalated between Paneth cells from WT and G4 *mTR*^*−/−*^ crypts (*n*=6); *P<0.05. All error bars reflect s.e.m., and *P* values reflect unpaired two-tailed Student's *t*-tests.

We examined CBC marker gene expression^18^ in successive generations of *mTR-*deficient mice, (rather than *mTert* mutants, to avoid perturbing potential non-telomeric roles of mTert (ref. 19)). Expression of the Wnt pathway target genes *Ascl2* and *Sox9* were decreased in late-generation (generation 4, or G4) *mTR*^*−/−*^ mice (Fig. 1a,b). Minimal changes in G2 mice indicate that the decreased expression is caused by dysfunctional telomeres rather than telomerase deficiency *per se*. Wnt ligands produced by stromal and Paneth cells provide support essential to CBC function^20^,^21^. Because *Ascl2 and Sox9* are direct targets of the Wnt signalling pathway, we hypothesized that the diminished expression in G4 *mTR*^*−/−*^ crypts reflects a defect in the Wnt pathway caused by telomere dysfunction. Crypt epithelium and underlying stroma were each isolated from wild type (WT) and G4 mice (Supplementary Fig. 1) and used for messenger RNA expression profiling. Indeed, among the sets most significantly downregulated in mutant crypts are those with genes similarly downregulated on deletion of the Wnt signalling mediator β-catenin, or altered with perturbation of other Wnt pathway components (Fig. 1c,d; Supplementary Figs 2 and 3b,d; Supplementary Table 1). Altered expression was confirmed by quantitative PCR for a number of the Wnt pathway and target genes, including *Wnt3*, *Lrp6 and Lgr5*, the last of which is not only a Wnt target and CBC marker, but also a key Wnt receptor component (Fig. 1d).

**Figure 2:**
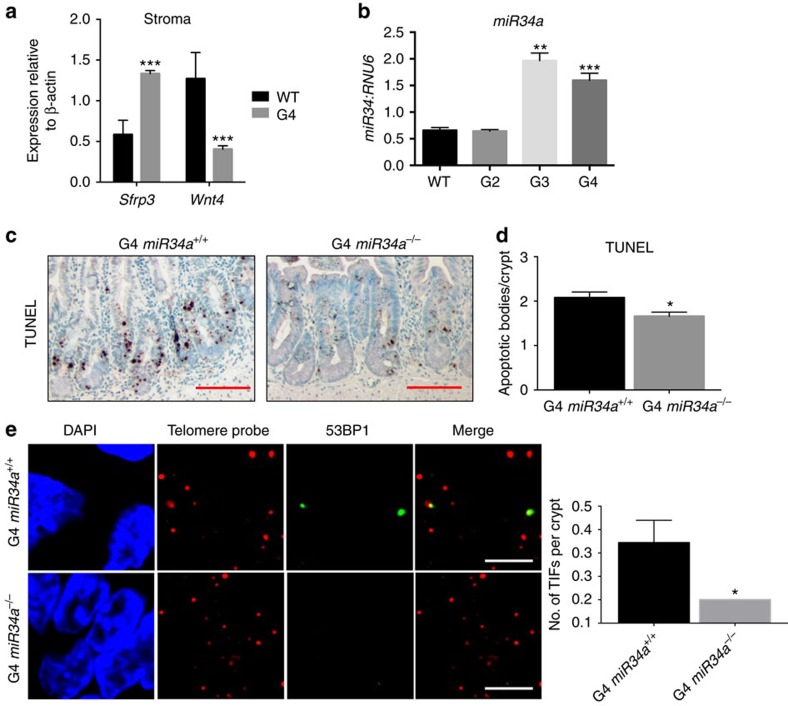
G4 *mTR*^*−/−*^ mice have reduced expression of pro-Wnt pathway genes in ileal crypts and stroma. (**a**) Quantitative reverse transcription PCR (qRT-PCR) analyses of gene expression for the Wnt ligand (*Wnt4*) and Wnt receptor inhibitor (*Sfrp3*) in WT and G4 *mTR*^*−/−*^ stroma (*n*=4). (**b**) qRT-PCR of *miR34a* in WT, G2, G3 and G4 *mTR*^*−/−*^ crypts (*n*=4 for WT, G2, G3; *n*=7 for G4). (**c**) Representative images showing apoptotic changes by TUNEL staining of ileum of G4 *mTR*^*−/−*^ *miR34a*^*+/+*^ mice and G4 *mTR*^*−/−*^ *miR34a*^*−/−*^mice. Scale bar, 50 μm. (**d**) Quantitation of TUNEL-positive apoptotic bodies per crypt (*n*=6). (**e**) Representative images showing reduced telomere dysfunction as measured by TIFs in G4 *mTR*^*−/−*^ *miR34a*^*−/−*^mice compared to littermate G4 *mTR*^*−/−*^ *miR34a*^*+/+*^mice (*n*=4). Quantitation of TIFs (right). Scale bar, 5 μm. All error bars reflect s.e.m., and *P* values reflect unpaired two-tailed Student's *t*-tests, with *P<0.05, **P<0.005 and ***P<0.0005.

Some degree of Wnt pathway gene downregulation may stem from CBC losses, but these cannot explain most of our observations. In particular, staining for lysozyme-positive Paneth cells revealed neither loss of these cells nor of interposed cells (Fig. 1e–g), where CBCs normally reside. Thus, diminished expression of CBC marker genes apparently reflects a change in state, rather than presence, of CBCs. Furthermore, the decline in *Wnt3* transcripts (Fig. 1d) cannot be explained by cell loses because Paneth cells are the only intestinal source of Wnt3 (ref. 21). Additionally, apoptosis is not significantly elevated in the stroma but changes consistent with decreased Wnt pathway activity were also found, including upregulation of SFRP Wnt antagonists (Fig. 2a; Supplementary Fig. 3a,c). There were also no significant changes in the expression of genes associated with quiescent ‘+4' ISCs, including *Bmi1, mTert, Hopx* and *Lrig1* (Supplementary Fig. 2c), consistent with maintenance of Wnt-independent gene expression. Collectively, the data from epithelium and stroma indicate broad Wnt pathway suppression in G4 *mTR*^*−/−*^ intestinal tissues.

Based on defects in G1 *mTert*^*−/−*^ but not G1 *mTR*^*−/−*^ mice, it was suggested^15^ that Tert but not TR regulates Wnt signalling, independent of its telomere-lengthening function. This idea has been questioned^22^,^23^, and moreover is quite different from our findings. First, our mice are genetically deficient for *mTR* and not *mTert,* and indeed *mTert* transcripts were not decreased in G4 *mTR*^*−/−*^ crypts (Supplementary Fig. 2c). Second, suppression of Wnt pathway gene expression progressed with successive generations (Fig. 1 and Supplementary Fig. 4b,c), confirming that telomere dysfunction, rather than telomerase deficiency *per se* causes the suppression.

### miR34a contributes to diminished Wnt pathway gene expression

We considered how Wnt pathway components are broadly downregulated in *mTR*^*−/−*^ mutants. Dysfunctional telomeres elicit p53-dependent DNA damage responses, and p53-activated upregulation of *miR34a* has recently been implicated in the suppression of multiple Wnt pathway genes^24^. Indeed, we detected p53 activation (as measured by p21 expression) and a significant increase in *miR34a* expression in *mTR*^*−/−*^ crypts (Fig. 2b; Supplementary Fig. 4a). Further, miRNA profiling indicated miR34a was the most upregulated canonical miRNA in mutant epithelium (Supplementary Table 2). We therefore tested interbred mice with genetic deficiencies in *miR34a* and *mTR* (Supplementary Fig. 5a) to test the hypothesis that miR34a loss would rescue defects associated with telomere dysfunction. Consistent with this idea, significantly more G4 mice survived to weaning at 3 weeks of age if they lacked miR34a (Supplementary Fig. 5b). Moreover, such G4 *mTR*^*−/−*^ *miR34a*^*−/−*^ mice exhibited significantly decreased crypt apoptosis, improved telomere capping as demonstrated fewer telomere-dysfunction-induced foci (TIFs; measured by colocalization of telomeres with 53BP1 (ref 25)), and a trend towards fewer anaphase bridges, indicative of fewer telomere–telomere fusions (Fig. 2c–e; Supplementary Fig. 5c). In addition, miR34a inhibition in cultured intestinal organoids derived from *mTR*^*−/−*^ mice restored crypt budding and expression of the Wnt pathway target genes *Lgr5* and *Trf2* (Supplementary Fig. 6, and see below). To extend published evidence for targeting of Wnt pathway genes by miR34a (Supplementary Table 3), we examined regulation of *Lgr4*, which cooperates with *Lgr5* to support CBCs (ref. 26). miR34a deletion increased expression in G4 fibroblasts of a luciferase reporter fused to the *Lgr4* 3′UTR, containing four miR34a target sequences, illustrating suppression of the Wnt pathway target by miR34a in G4 *mTR*^*−/−*^ cells (Supplementary Fig. 7).

### Rescue telomere defects by Wnt pathway agonists

**Figure 3:**
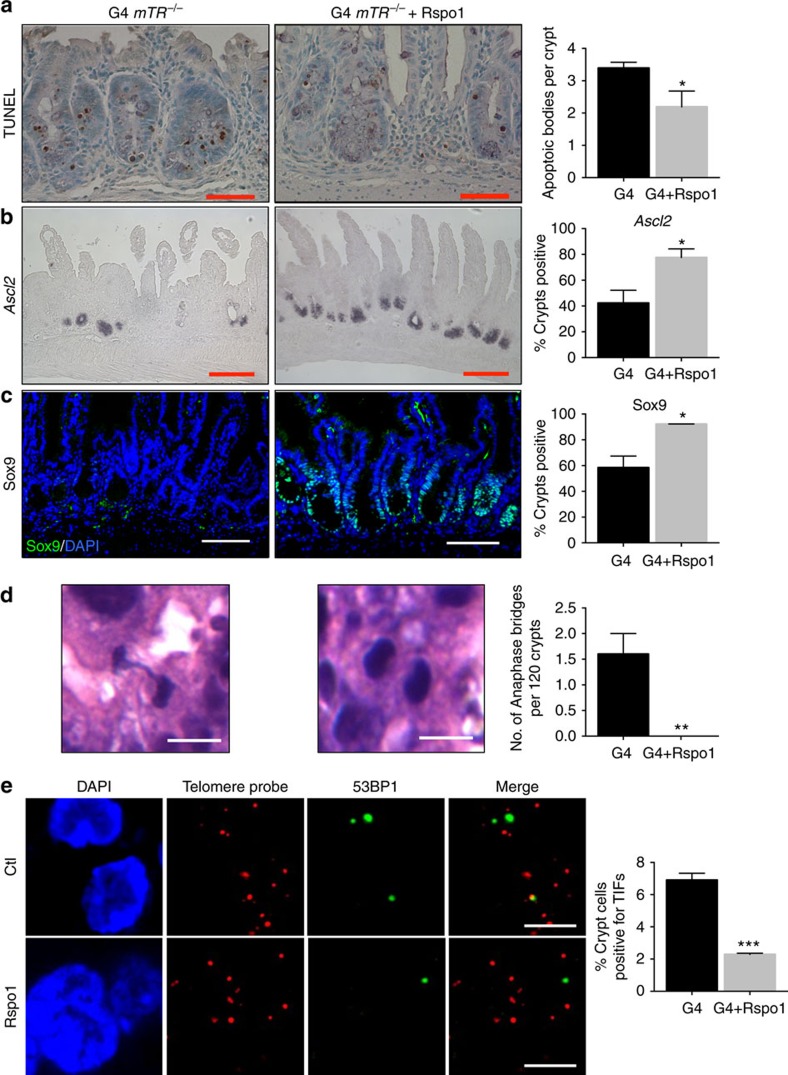
Enhanced Wnt signalling rescues G4 *mTR*^*−/−*^ crypt dysfunction and telomere uncapping *in vivo*. (**a**) Representative images showing apoptotic changes by TUNEL staining in intestinal crypts of G4 *mTR*^*−/−*^ mice treated for 8 days with subcutaneous injections of R-spondin1 (Rspo1) (*n*=4 Rspo1-treated and 5 littermate controls). Scale bars, 50 μm. (**b**) Representative image showing *Ascl2* transcripts differences in Rspo1-treated and control G4 *mTR*^*−/−*^ mice (*n*=3). Scale bars, 100 μm. (**c**) Representative images showing Sox9 protein differences in Rspo1-treated and control G4 *mTR*^*−/−*^ mice (*n*=4 control and 3 Rspo1-treated). Scale bars, 50 μm. (**d**) Representative images showing reduced telomere dysfunction as measured by anaphase bridges in Rspo1-treated G4 *mTR*^*−/−*^ mice (left, untreated; middle, Rspo1-treated). Quantitation of anaphase bridges (right). 120 crypts per mouse from 5 control G4 mice and 4 Rspo1-treated G4 mice were surveyed for anaphase bridges. Scale bars, 10 μm. (**e**) Representative images and quantitation (right) showing reduced telomere dysfunction as measured by TIFs in Rspo1-treated G4 *mTR*^*−/−*^ mice (*n*=3). Scale bars, 5 μm. All error bars reflect s.e.m., and *P* values reflect unpaired two-tailed Student's *t*-tests, with *P<0.05, **P<0.005 and *** *P*<0.0005.

**Figure 4:**
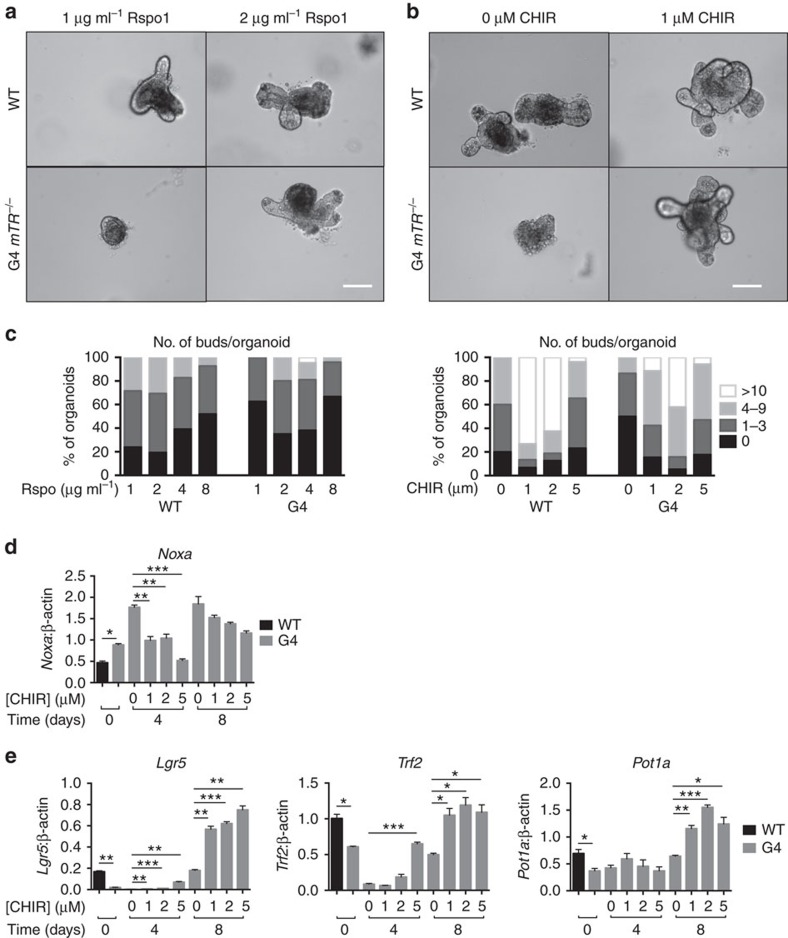
Wnt pathway agonists rescue survival and morphology of cultured G4 *mTR*^*−/−*^ intestinal organoids. (**a**,**b**) Representative micrographs showing intestinal organoids from WT and G4 *mTR*^*−/−*^ mice cultured under standard conditions (1 μg ml^−1^ Rspo1) or augmented with elevated levels of (**a**) Rspo1 or (**b**) the glycogen synthase kinase 3 inhibitor CHIR99021. Under standard culture conditions, WT organoids are long-lived and appear as roughly spherical epithelium surrounding a central cavity representing the lumen, and have crypt buds radiating outward from their peripheries. G4 *mTR*^*−/−*^ organoids were prone to degeneration and had fewer crypts under basal conditions, but could be rescued with slightly elevated Rspo1 or CHIR99021. Scale bars, 100 μm. (**c**) The number of buds per organoid was quantified and grouped accordingly (0, 1–3, 4–9, >10 buds per organoid), and expressed as a percentage of all organoids. Note that the well-established inhibition of crypt budding by supraphysiologic levels of Wnt pathway activity is shifted to higher doses of Rspo and CHIR in the G4 organoids, consistent with a basal Wnt pathway defect. (**d**) Quantitative reverse transcription PCR (qRT-PCR) measurement of *Noxa* transcripts, a mediator of p53-activated apoptosis, in WT and G4 *mTR*^*−/−*^ crypts at *t*=0, and treated with increasing doses of CHIR99021 at days 4 or 8 (*n*=3); *P<0.05, **P<0.01 and ***P<0.0001. (**e**) Dose- and time-dependent increase of *Lgr5, Tfr2* and *Pot1a* transcript expression as measured by qRT-PCR in WT and G4 *mTR*^*−/−*^ crypts cultured with CHIR99021 for 4 or 8 consecutive days (*n*=3). For all panels, *P<0.05, **P<0.005 and ***P<0.0005. Also, note that miR34a inhibition similarly rescued organoid budding, Lgr5 and Trf2 expression (Supplementary Fig. 6). All error bars reflect s.e.m., and *P* values reflect unpaired two-tailed Student's *t*-tests.

We next asked whether Wnt pathway agonists could ameliorate pathology by restoring the niche environment. Indeed, subcutaneous injection of Rspo1, which selectively potentiates intestinal Wnt signalling via the Lgr5 co-receptor^27^, suppressed apoptosis and led to elevated *Ascl2* transcript and Sox9 protein levels (Fig. 3a–c). Furthermore, crypt apoptosis and *Ascl2* expression were rescued in G3 *mTR*^*−/−*^ mice fed chow containing lithium, which is a glycogen synthase kinase 3 inhibitor^28^, and this rescue persisted for at least 44 days (Supplementary Figs 8 and 9). As a complementary approach, we tested effects of Rspo1 and the selective glycogen synthase kinase 3 inhibitor CHIR99021 (ref. 29) on G4 *mTR*^*−/−*^ intestinal organoid cultures. Both Rspo1 and CHIR99021 yielded dose-dependent improved mutant organoid crypt morphology, and CHIR99021 suppressed transcripts of *Noxa* (a mediator of p53-induced apoptosis) and rescued *Lgr5* transcript levels (Fig. 4a–e). In sum, these *in vivo* and *in vitro* data establish roles for Wnt pathway agonists in rescuing abnormalities in late-generation *mTR*^*−/−*^ crypts related to morphology, apoptosis and CBC-related gene expression.

Wnt pathway activity is known to enhance intestinal epithelial cell proliferation, and we observed increased Ki67-positive cells in the transit amplifying, but not crypt bases, of Rspo1 and lithium-treated G4 *mTR*^*−/−*^ mutants (Supplementary Fig. 10). Without telomerase activity, enhanced proliferation should only exacerbate telomere shortening and dysfunction, making rescue seemingly paradoxical. We therefore investigated whether rescue might be associated with improved telomere capping. We scored TIFs and found a significant decrease in the number in crypts of G4 *mTR*^*−/−*^ mice treated with Rspo1, as well as decreased anaphase bridges, indicating fewer telomere fusions (Fig. 3d,e). Telomere lengths in crypt epithelial cells of lithium or Rspo1-treated G4 *mTR*^*−/−*^ mice were not longer than those of untreated G4 *mTR*^*−/−*^ mice (Supplementary Fig. 11), ruling-out telomerase-independent lengthening. In fact, telomeres appeared to shorten in lithium-treated mice, consistent with enhanced proliferation due to improved telomere capping. In the complete absence of telomerase, additional telomere shortening would presumably lead eventually to stem cell exhaustion. Nonetheless, long-term (44 day) treatment with lithium maintained telomere capping, suppressed apoptosis and anaphase bridges, and enhanced *Ascl2* expression (Supplementary Fig. 9). Therefore, Wnt pathway agonists can restore capping of short telomeres without net lengthening in the absence of telomerase.

Telomere capping depends not only on telomere length but also on several key shelterin proteins, including Trf2 and Pot1a (ref. 30). *Trf2* was shown recently to be upregulated by the Wnt pathway in human cells and murine intestinal tissue, and elevated Trf2 can rescue uncapped telomeres in senescing human fibroblasts and following β-catenin inhibition in cancer cells^31^,^32^. We analysed published data sets and found additional evidence that shelterin genes are regulated by Wnt, including APC- and β-catenin-dependent regulation of *Trf2*, and TCF4 binding to *TRF1, TRF2* and *POT1* (but not *TPP1* and *RAP1*) in humans (Supplementary Fig. 12)^33^,^34^. Consistent with these observations, levels of *Trf1*, *Trf2, Pot1a/b* (and not *Tpp1* and *Rap1*) transcripts were reduced significantly in late-generation *mTR*^*−/−*^ crypts (Supplementary Figs 4c and 12a). Moreover, CHIR99021 provided dose-dependent upregulation of *Trf2*, *Pot1a*, *Tin2* and *Trf1* transcripts in G4 crypt cultures (Fig. 4e and Supplementary Fig. 13). Similar results were obtained in human patient-derived fibroblasts with premature telomere uncapping caused by telomerase deficiency (dykeratosis congenita, DC) or telomere replication defects (Werner syndrome; Supplementary Fig. 14). Thus telomere capping via Wnt pathway activation may be explained by upregulation of shelterin components.

Our study reveals that loss of telomere capping can lead to broad suppression of Wnt pathway activities in both intestinal epithelium and stroma, and moreover, that enhancement of Wnt pathway activity can rescue telomere capping, apoptosis and the ISC gene expression program. Importantly, components of the Wnt pathway are themselves pathway targets (for example, *Lgr5*, encoding a Rspo1-responsive component of the Wnt receptor complex)^18^, providing a mechanism by which the effects of Wnt pathway agonists can be amplified to restore the pathway. Improved telomere capping is not explained by telomere lengthening, but rather is associated with restoration of expression of key shelterin genes, including *Trf2* and *Pot1a*. Whether Wnt pathway agonists sensitize or protect intestine from ionizing radiation is debated^14^,^35^,^36^. Regardless, upregulation of shelterin proteins would be expected to have a salutary effect for shortened telomeres but not radiation-induced genome-wide breaks, consistent with the clear benefits of enhanced Wnt pathway activity in *mTR*^*−/−*^ mutants. Our findings raise the possibility that disorders in which telomere dysfunction plays a pathogenic role (for example, DC or ulcerative colitis)^37^,^38^,^39^ might be treated by Wnt pathway agonists. The haematopoietic system is often severely affected in patients with telomere diseases, and since canonical and noncanonical Wnt signalling play complex roles in haematopoietic homeostasis^40^,^41^,^42^, further studies are warranted to establish effects Wnt agonists have on the haematopoietic system in the setting of telomere dysfunction. However, lithium has been used for many decades to treat bipolar disorder, and leucocyte telomere lengths and TERT expression in treated patients positively correlate with duration of therapy^43^,^44^. This therapeutic approach might raise concern given that hyper-activation of Wnt signalling can drive cancer, but a recent large-scale case-control study found no association between long-term lithium use and risk of colorectal adenocarcinoma^45^. Furthermore, the elevated cancer rates of DC patients indicate that excessive telomere shortening due to telomerase deficiency is itself carcinogenic, and therefore improved telomere capping may actually suppress cancer in such individuals. Wnt agonists may be of particular benefit in patients with such telomere diseases because they may not only upregulate key shelterins and improve telomere capping at any given telomere length (as our current study demonstrates), but due to their ability to effect *TERT* upregulation^16^,^17^ they may also improve telomere capping via lengthening.

## Methods

### Study design

Histologic comparisons of WT, G2, G4 *mTR*^*−/−*^ and G4 *mTR*^*−/−*^ *miR34a*^*−/−*^ mice were made between groups each having similar average age, and all mice were younger than 12 months. RNA samples for microarray analysis and quantitative reverse transcription PCR validation were obtained from WT and G4 *mTR*^*−/−*^ mice aged 7–8 months. For each of the lithium and Rspo1 treatment experiments, littermates were divided equally into the control and treatment groups. Comparisons were made between littermates to minimize differences in inherited telomere lengths. For cultured crypts experiments, G4 *mTR*^*−/−*^ mice aged 3 months or younger were used because survival of crypts dropped precipitously beyond this age.

### Mice

All mice were on the C57BL/6 J background, and *mTR*^+/−^ mice were crossed to generate G1 *mTR*^*−/−*^ mice, which were crossed to generate G2 mice, and so forth^9^. *mTR* mutant mice were obtained from the laboratory of R. DePinho and backcrossed 12 times onto the C57Bl/6 J background, and *miR34a*^*−/−*^ (B6.Cg-Mir34a^tm1Lhe^/J) mice were obtained from Jackson Laboratory. Comparisons were made between age- and sex-matched mice except in the long-term lithium treatment experiment where sex-matching was not possible (Supplementary Fig. 9). In addition, sex-matching was not always possible for comparisons between G4 *mTR*^*−/−*^ *miR34a*^*−/−*^ and G4 *mTR*^*−/−*^ *miR34a*^+/+^ mice. Mice for the microarray experiment were females and ranged from 6 to 8 months old. Mice for the Rspo1 and lithium treatments were males and ranged from 6 to 8 months old. Crypts were isolated from mice 1 to 3 months of age for organoid culture experiments. The breeding scheme for generating *miR34a*^*−/−*^ onto the different generations of *mTR*^*−/−*^ mice is outlined in Supplementary Fig. 5a. All studies are approved by the University of Pennsylvania Institutional Animal Care and Use Committee. The mice were housed in a standard animal care room with 12:12-h light–dark cycle with free access to food and water. For the short-term 10-day lithium treatment, mice were given an *ad libitum* lithium-chow diet of 0.212% lithium chloride-supplemented chow (Harlan Teklad, Madison, WI, USA) for 3 days, followed by 0.4% lithium chloride-supplemented chow for 7 days. For the long-term 44-day lithium treatment, mice were given 4 consecutive cycles of 0.212% lithium-chow for 4 days, followed by 0.4% lithium-chow for 7 days. Lithium-chow-fed mice were given a supplemental source of drinking water containing 1.5% (w/v) sodium chloride to counteract potential toxicities of lithium. For Rspo1 experiments, mice are injected at 4 μg Rspo1 (in PBS, or PBS alone for controls) per gram body weight subcutaneously daily for 8 days. Rspo1 was expressed and purified as described^46^. Briefly, HEK293T cells stably transfected with an hRspo1 expression vector were cultured in suspension, protein was precipitated from the culture medium with ammonium sulfate, purified by column chromatography, dialyzed into PBS, 0.22 μM filtered, and stored at −80 °C.

### Tissue histology

Standard 5-μ sections were cut from formalin-fixed and paraffin-embedded samples. *In situ* hybridization for *Ascl2* was as described^18^. Briefly, for *in situ* hybridization, formalin-fixed and paraffin-embedded samples were deparaffinized, rehydrated, treated with HCl, digested in proteinase K solution, refixed with formaldehyde, treated in acetic anhydride solution, and hybridized with a digoxigenin labelled RNA probe specific for *Ascl2*. After washes with 2 × saline-sodium citrate (SSC) and 50% formamide/2 × SSC, sections were incubated overnight at 4 °C with alkaline phosphatase-conjugated anti-digoxigenin antibodies (Roche #11093274910 at a 1:2,000 dilution, that is, 0.375 units per ml), and developed using nitro blue tetrazolium/5-bromo-4-chloro-3-indolyl-phosphate (NBT/BCIP) solution. For immunofluorescent detection of Sox9, sections were treated as follows, at room temperature unless stated otherwise. Sections were deparaffinized by heating to 60 °C for 15 min, washed 3 × 5 min in xylene, rehydrated in a series of ethanol solutions (100%, 95%, 90% and 70% ethanol), washed in water and in 0.1% Tween-20 for 1 min each, steamed in antigen unmasking solution (Vector Laboratories H-3300) for 15 min, washed in phosphate-buffered saline containing 0.2% Tween-20 (PBST) for 5 min, blocked in 4% BSA in PBST for 30 min, incubated with rabbit anti-Sox9 antibodies (Millipore AB5535 at 1.7 μg ml^−1^) overnight at 4° in 1% BSA in PBST, washed three times for 5 min in PBST, incubated with Alexa 488-conjugated goat anti-rabbit antibody (Invitrogen A-11034 at 2.5 μg ml^−1^) for 2 hours, washed three times for five minutes in PBST including 0.2 μg ml^−1^ 4,6-diamidino-2-phenylindole (DAPI) in the penultimate wash, and mounted in ProLong Gold antifade reagent (Thermo Fisher Scientific). For immunohistochemical detection of Ki67, sections were deparaffinized, rehydrated and subjected to antigen unmasking in the same fashion as for Sox9 staining, and were then treated with 2.25% hydrogen peroxide for 15 min, washed in water and PBST for 5 min each, blocked with Avidin D reagent (Vector Laboratories SP-2001), washed in PBST, blocked with 4% BSA in PBST for 30 min, incubated with rabbit anti-Ki67 antibody (Abcam Ab16667 at 1:100), washed three times for 5 min in PBST, incubated with biotinylated anti-rabbit antibody (Vector Laboratories BA-1000 at 7.5 μg ml^−1^) for 30 min, washed in PBST 3–5 min, stained with the Vector Laboratories ABC and DAB reagents (PK-6200 and SK-4100), counterstained with Gill #2 haematoxylin (Thermo Fisher Scientific), dehydrated in an ethanol series (70–100%), cleared in xylene, and mounted in Cytoseal XYL (Thermo Fisher Scientific 8312-4). Terminal deoxynucleotidyl transferase dUTP nick-end labeling (TUNEL) assays were performed using the ApopTag Peroxidase In Situ apoptosis Detection Kit (Chemicon). Images within each set of staining were taken with identical settings on a Nikon Eclipse E600 microscope. Images were taken with identical settings, and brightness/contrast was adjusted post-capture in a linear fashion and equally for all samples. 120 crypts per mouse from haematoxylin and eosin stained sections were surveyed for the presence of anaphase bridges.

### TIF and telomere quantitative fluorescence *in situ* hybridization analyses

Tissue sections were hybridized with a Cy3-labelled PNA telomere repeat probe (Panagene, (5′-CCCTAA-3′)_3_) and anti-53BP1 antibodies (Novus, NB100-304) as described with slight modifications^47^. Briefly, formalin-fixed and paraffin-embedded tissue sections were deparaffinized with xylene, rehydrated with 95, 85 and 70% ethanol series, rinsed with H_2_O and 0.1% Tween-20, and subjected to antigen retrieval by covering with sodium citrate buffer (Vector labs, H-2200) and a cover slide and then steaming for 15 min. Sections were then washed with PBST (0.2% Tween-20 in PBS), and then blocked in 4% BSA/PBST for 30 min at 37 °C, incubated with rabbit anti-53BP1 antibodies (10 μg ml^−1^) for 2 h at 37 °C, washed with PBST, incubated and Alexa Fluor 488-conjugated goat anti-rabbit antibodies (Invitrogen, A-11034, 4 μg ml^−1^) for 1 h at 37 °C. After washing with PBST, slides were fixed with 4% paraformaldehyde in PBS at room temperature for 20 min, followed by quenching of the formaldehyde with 0.25 mM glycine. The sections were subsequently dehydrated with ethanol and air dried. Cy3-conjugated telomere-specific PNA probe was applied in hybridization mix (per 100 μl, mix 70 μl of freshly deionized formamide, 15 μl PNA buffer (80 mM Tris-Cl pH 8, 33 mM KCl, 6.7 mM MgCl_2_, 0.0067% Triton X-100), 10 μl 25 mg ml^−1^ acetylated BSA, 5 μl 10 μg ml^−1^ PNA probe). Sections were covered with a glass coverslip, denatured at 83 °C for 4 min on a heating block and incubated in the dark in a humidified chamber overnight at room temperature. Slides were then washed, blocked and subsequently incubated with Alexa Fluor 488-conjugated donkey anti-goat antibodies (Invitrogen, A-11055, 4 μg ml^−1^), stained with DAPI and mounted in ProLong Gold antifade reagent (Invitrogen, P36934). Confocal images were obtained with a Nikon Ti-U inverted microscope with CSU-10 spinning disk confocal head (Spectral Boralis) using a Nikon Plan APO60x/1.4 lens and Hamamatsu Orca-ER camera. The Cy3 laser was held at a constant intensity to capture all images. Images from mice treated with lithium and Rspo1 were obtained in 16-bit grey scale and 8-bit RGB24 formats, respectively. To measure telomere lengths, quantitative image analysis was performed on confocal images using Media Cybernetics and ImageProPlus 7.01 software. The DAPI images were used to define the nuclear area in which to measure telomere intensities. Images were first deconvoluted, and nuclear outlines of interest were drawn and applied as a mask onto the Cy3 images. Cy3 images were also deconvoluted, and telomere intensities within each nucleus were obtained (for 16-bit images applied filters were: 1,000–65,535 pixel intensity threshold and area larger than 11.25sq. microns; for 8-bit images applied filters were: density/intensity ratio threshold of 0-254). Statistical analyses of differences in telomere lengths between samples were performed using the Mann–Whitney *U*-test.

### Isolation of intestinal crypt and stromal cells

Ileum of 8 cm was longitudinally cut and rinsed in cold PBS, followed by firm scraping of intestinal villi with haemacytometer coverslip, and incubation in 10 ml 30 mM EDTA and 1.5 mM DTT in PBS on ice for 20 min. The intestine was transferred to 10 ml 30 mM EDTA in PBS at 37 °C for 8 min, followed by 3 rounds of shaking per second for 30–40 s. The supernatant was centrifuged at 200 g for 1 min at 4 °C, followed by passage through a 70 μm cell strainer. The strained supernatant was spun down again at 130 g for 1 min at 4 °C to deplete single cells, and the crypt pellet was collected for RNA extraction. The remaining intestinal tissue is scraped with a coverslip to remove residual epithelial cells and is mechanically homogenized with a TissueRuptor (Qiagen) before RNA extraction.

### RNA extraction

All RNA extractions were performed with the miRNeasy Kit (Qiagen). RNA quality was confirmed with Nanodrop spectrophotometry or Agilent Bioanalyzer.

### Quantitative RT–PCR

Reverse transcription was performed with miScript II RT Kit (Qiagen) according to the manufacturer, using miScript HiFlex buffer. Real-time PCR was performed on a Roche LightCycler 480 using SYBR Green JumpStart Taq ReadyMix (Sigma). Reactions (10 ml) were performed in triplicate, as follows: 10 min at 95 °C, 45 cycles of 15 s at 95 °C, 30 s at 59 °C, 30 s at 72 °C. Melt curve and gel electrophoretic analyses were performed to verify specific target amplification. Amplification from experimental samples was normalized to standard curves that were made from dilutions of pooled samples. Cp values from each amplification curve were computed by the second-derivative method, the messenger RNA expression levels were normalized to that of β-actin, and the mature miRNA expression levels were normalized to that of RNU6. Primer sequences are provided in Supplementary Table 4. Student's *t*-tests were used for comparisons, except that a two-way analysis of variance (multiple comparisons) was used for statistical analyses of human fibroblasts, comparing the gene expression at each dose of CHIR99021 to no drug within each genotype.

### Microarray protocol and analysis

Microarray experiments were conducted by the University of Pennsylvania Molecular Profiling Facility, including quality control tests of the total RNA samples by Agilent Bioanalyzer and Nanodrop spectrophotometry. Crypt and stromal RNA was obtained, respectively, from three or two WT and three or four G4 *mTR*^*−/−*^ mice, with samples from each mouse analysed on an individual array. All protocols were conducted as described in the Ambion WT Expression Manual and the Affymetrix GeneChip Expression Analysis Technical Manual, using 250 ng of total RNA for first-strand cDNA synthesis. cDNA yields ranged from 9.4 to 10.7 μg, and 5.5 μg of labelled cDNA was hybridized to Mouse Gene 1.0ST GeneChips, stained with streptavidin–phycoerythrin, and visualized with a GeneChip 3000 7G scanner. Affymetrix probe intensity (.cel) files were imported into Partek Genomics Suite (v6.6, Partek Inc., St Louis, MO, USA) where RMA normalization was applied. The resulting log_2_-transformed intensities were filtered to exclude the IDs corresponding to technical controls, and analysed for differential expression using SAM (Significance Analysis of Microarrays, samr v2.0, Stanford University^48^), generating *q*-values (False Discovery Rate) and fold change for each Transcript ID. For the miRNA microarray, crypt RNA obtained from three G4 *mTR*^*−/−*^ mice (same as that used in messenger RNA microarray), with samples from each mouse analysed on an individual array. Amplification steps were performed with the Genisphere FlashTag (miRNA) kit and the Affymetrix GeneChip miRNA 3.0 Array chips were used. Affymetrix probe intensity (.cel) files were imported into Partek Genomics Suite (v6.6, Partek Inc., St. Louis, MO, USA) where RMA normalization was applied. The resulting log_2_-transformed intensities were filtered to exclude the IDs corresponding to technical controls, and analysed for differential expression using SAM, generating *q*-values (False Discovery Rate) and fold change for each Transcript ID.

### Heat map and gene set enrichment analysis (GSEA)

Heat maps were generated with Multiple Experiment Viewer (MeV; ref. 49) v4.8.1 from the Dana-Farber Cancer Institute. GSEA (ref. 50) v2.0.13 from the Broad Institute was used to identify significant enrichments in the microarray data. The C2 collection containing 4850 curated gene sets from the Molecular Signature Database (MSigDB) v3.1 was used to identify enriched pathways in WT versus G4 *mTR*^*−/−*^ crypts. Chip2Chip was used to translate gene identifiers from the C2 collection to the Affymetrix Mouse Gene 1.0ST probeset IDs. GSEA was performed as follows: log_2_-transformed RMA values from each individual sample were used for the expression data set. Parameter details are as follows: Permutations: 1,000, ‘Collapse data set'=FALSE, Permutation type=gene_set, Max size=5,000, Min size=1. GSEA was also performed using user-defined gene sets from the transcriptomic profile of ISCs or downregulated genes in Lgr4/5 KO crypts, described as follows. 361 genes out of 379 genes defined as the transcriptomic profile of Lgr5+ ISCs on the Affymetrix platform^51^ matched the Affymetrix Mouse Gene 1.0ST platform that was used for measurements, and these were used to generate the heat map and GSEA enrichment plot. We also used the 125 genes out of 135 genes defined as the ISC transcriptome^18^ that matched our microarray platform. For the gene set describing downregulated genes in Lgr4/5 KO crypts^26^, 259 out of 306 genes from the array platforms matched the Affymetrix platform and were used in GSEA. GSEA was performed for proliferating (PD32) and replicatively senescent (PD88) IMR90 cells using publicly available Gene Expression Omnibus (GEO) data set GSE36640 (ref. 52). Chip2Chip was used to translate gene identifiers from the C2 collection to the Affymetrix Human Genome U133 Plus 2.0 Array. GSEA was performed as follows: raw data set values from each individual sample were used for the expression data set. Parameter details are as follows: Permutations: 1,000, ‘Collapse data set'=FALSE, Permutation type=gene_set, Max size=10,000, Min size=5, and 4721 out of 4722 C2 collection of gene sets from MsigDB v4.0 passed the criteria for analysis.

### Crypt culture

Intestinal crypts used for culturing were isolated from the proximal half of the small intestine as described^53^. Briefly, villi were scraped off the small intestinal lumen with a glass slide, and the remaining tissue was cut in 2–4 mm pieces, incubated with 30 ml of 30 mM EDTA in PBS for 15 min on ice, with occasional inversion. The tissues were allowed to settle by gravity and subsequently, the mixture was washed with ice cold PBS, and the supernatant was discarded. The wash/discard cycle was repeated for 10–15 rounds or until the supernatant was composed predominantly of crypts, which was filtered through a 70 μm cell strainer into a 50 ml centrifuge tube and pelleted via centrifugation at 300 g for 5 min. Isolated crypts were cultured in Matrigel (BD Biosciences) with advanced DMEM/F12 medium containing final and basal concentrations of 100 ng ml^−1^ noggin, 50 ng ml^−1^ mEGF and 1 μg ml^−1^ RSpo1 (higher levels of RSpo1 were used for some experiments as indicated). Approximately 200–500 crypts per 50 μl of Matrigel were plated per well in a pre-warmed 24-well plate and cultured at 37 °C in a 6% CO_2_ incubator with ambient O_2_. CHIR99021 (Tocris Biosciences) was prepared as a 2 mM stock solution in DMSO. Lentiviruses were made at the Wistar Vector Core. Anti-miRNA plasmids targeting miR34a (MZIP34a-PA-1) and control plasmids (MZIP000-PA-1) were obtained from System Biosciences. Organoids were cultured and established in 5 μM CHIR conditions for several passages before infection. Organoids embedded in Matrigel were counted and washed once with cold PBS, and the organoids and Matrigel were broken up by pipetting. Organoids were spun down at 300 g in a cold centrifuge and resuspended in culture media containing 5 μM CHIR. Lentiviruses were added to the organoid suspension at different multiplicities of infection (MOI of 25 or 50) for 4–6 h in a 37 °C incubator. After incubation, organoids were collected and spun down at 300 g, resuspended in Matrigel, plated, and grown at 5 μM CHIR for 2–3 days before 2 μg ml^−1^ puromycin selection.

### Cell lines

Primary human fibroblasts were obtained from the Coriell Institute. The two DC lines were: GM01774 (population doubling level (PD) 25; from 7-year-old male), and AG04646 (PD 21; from 11-year-old male). The three healthy control (WT) lines were: GM01786 (PD 25; 30-year-old mother of GM01774), GM00409 (PD ∼16; from 7-year-old male) and GM00323 (PD 23.6; from 11-year-old male). The Werner syndrome line was AG05229B (PD ∼30; from 25-year-old male), which was retrovirally transduced with pBABE-puro-hTERT, or with the empty pBABE-puro vector as a control. WT, *miR34a*^*−/−*^, sibling-matched G4 *mTR*^*−/−*^ *miR34a*^*+/+*^, G4 *mTR*^*−/−*^ *miR34a*^*−/−*^ mouse skin fibroblasts were cultured from ear clippings. Mycoplasma contamination testing was not performed on these cultures, but general testing of cell cultures in our laboratory has not revealed mycoplasma contamination. miR34a overexpression and control retroviral vectors (pMSCV-PIG-34a and pMSCV-PIG) were obtained from Joshua Mendell. *miR34a*^*−/−*^ fibroblasts were transduced with the retroviruses and selected with puromycin. Fibroblasts were cultured in DMEM with 15% FBS, with 1X penicillin/streptomycin/amphotericin-B at 37 °C in a 6% CO_2_ and 3% O_2_ atmosphere.

### miR34a target prediction and 3′UTR reporter assays

Several databases were used to predict miR34a target sites on candidate genes. Candidate genes are Wnt pathway genes that have not yet been experimentally tested as miR34a targets elsewhere in literature (Supplementary Table 3). Candidate genes were screened with microRNA.org (http://www.microrna.org/), mirdb.org (http://mirdb.org/miRDB/), TargetScan (http://www.targetscan.org/) and RNA22 (https://cm.jefferson.edu/rna22/Precomputed) to identify potential miR34a target sequences. The 3′UTR of *Lgr4* (NM_172671; +1 to +1728) was PCR amplified from genomic mouse tail DNA and subcloned into the *XhoI* and *NotI* sites downstream of renilla luciferase of the psiCHECK2 vector (Promega). Mouse skin fibroblasts were transfected with 500 ng of reporter construct with Fugene 6 transfection reagent (Promega). Cells were lysed 24 h after transfection and the luciferase activity was measured by the Dual-GLO luciferase assay (Promega).

### Statistics

Bar graphs are represented with s.e.m. error bars unless otherwise stated. *P* values were calculated with Prism. Unpaired *t*-tests assuming equal population s.d. were performed unless specified. Two-way analysis of variance was performed for Supplementary Fig. 14.

### Data availability

The gene expression profiling microarray data generated during the study have been deposited in Gene Expression Omnibus (GEO) with the accession code GSE90820 (https://www.ncbi.nlm.nih.gov/geo/query/acc.cgi?acc=GSE90820). The remaining data that support the findings of this study are available from the authors upon request.

## Additional information

**How to cite this article:** Yang, T.-L. B. *et al*. Mutual reinforcement between telomere capping and canonical Wnt signalling in the intestinal stem cell niche. *Nat. Commun.* **8,** 14766 doi: 10.1038/ncomms14766 (2017).

**Publisher's note:** Springer Nature remains neutral with regard to jurisdictional claims in published maps and institutional affiliations.

## Supplementary Material

Supplementary Information Supplementary Figures, Supplementary Tables and Supplementary References

Peer Review File
