# Supplementary material for: Mutual reinforcement between telomere capping and canonical Wnt signalling in the intestinal stem cell niche
Source: Nat Commun. 2017 Mar 17;8:14766. doi: 10.1038/ncomms14766 (PMC5357864; doi:10.1038/ncomms14766)
Supplement: Supplementary Information — Supplementary Figures, Supplementary Tables and Supplementary References [file ncomms14766-s1.pdf]

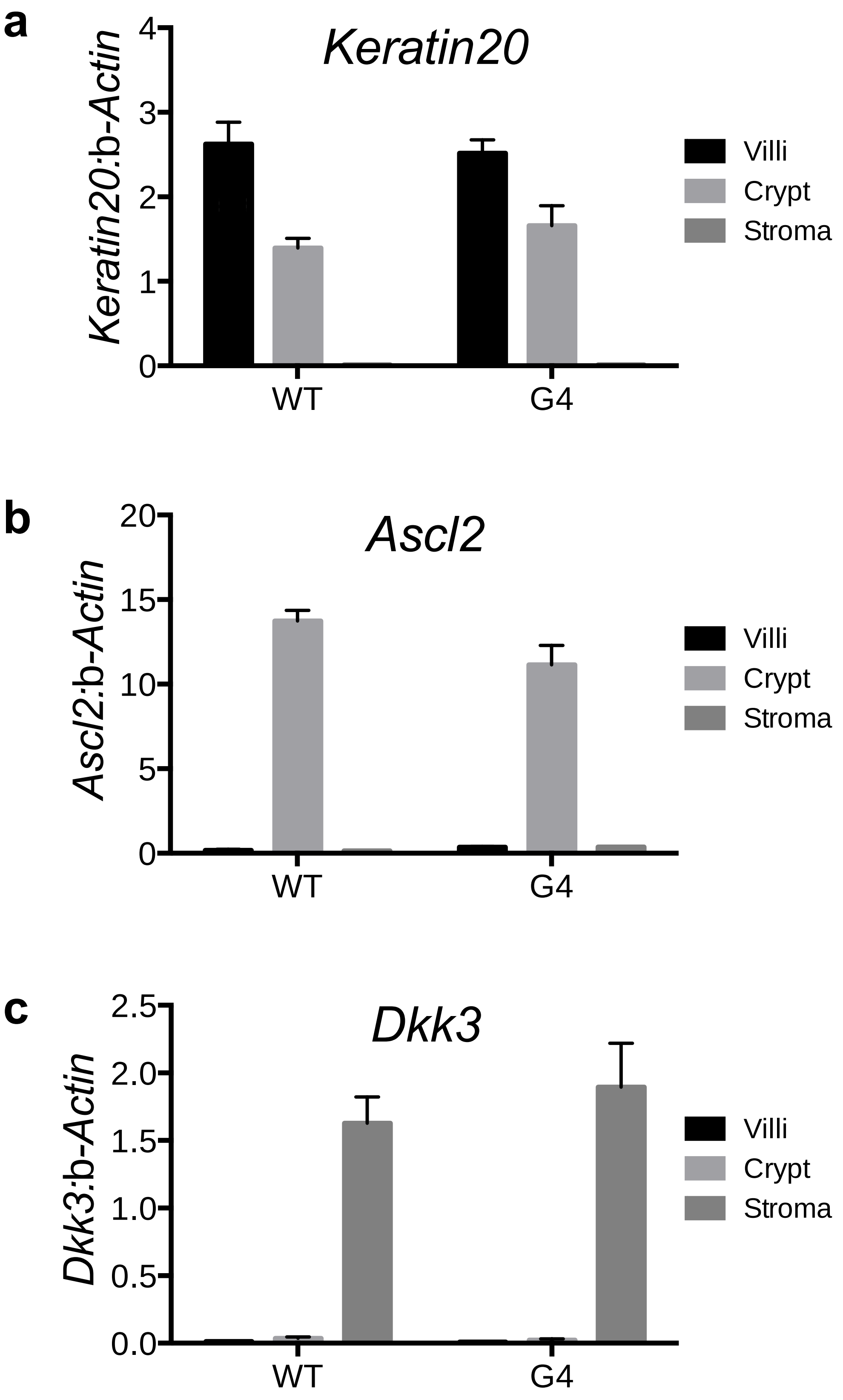
a
Keratin20
Keratin20:b-Actin
0
1
2
3
4
WT
G4
Villi
Crypt
Stroma
b
Ascl2
Ascl2:b-Actin
0
5
10
15
20
WT
G4
Villi
Crypt
Stroma
c
Dkk3
Dkk3:b-Actin
0.0
0.5
1.0
1.5
2.0
2.5
WT
G4
Villi
Crypt
Stroma

Supplementary Figure 1. Assessment of intestinal villus, crypt, and stromal purity by qRT-PCR. (**a-c**) The ileum from WT and G4 *mTR*$^{-/-}$ mice was separated into villus, crypt, and stromal fractions (as described in *Methods*), and the fractions were assessed for expression of genes restricted to the (**a**) epithelium (*Keratin20*), (**b**) crypt (*Ascl2*), and (**c**) stroma (*Dkk3*) (n = 3). Error bars are SEMs.

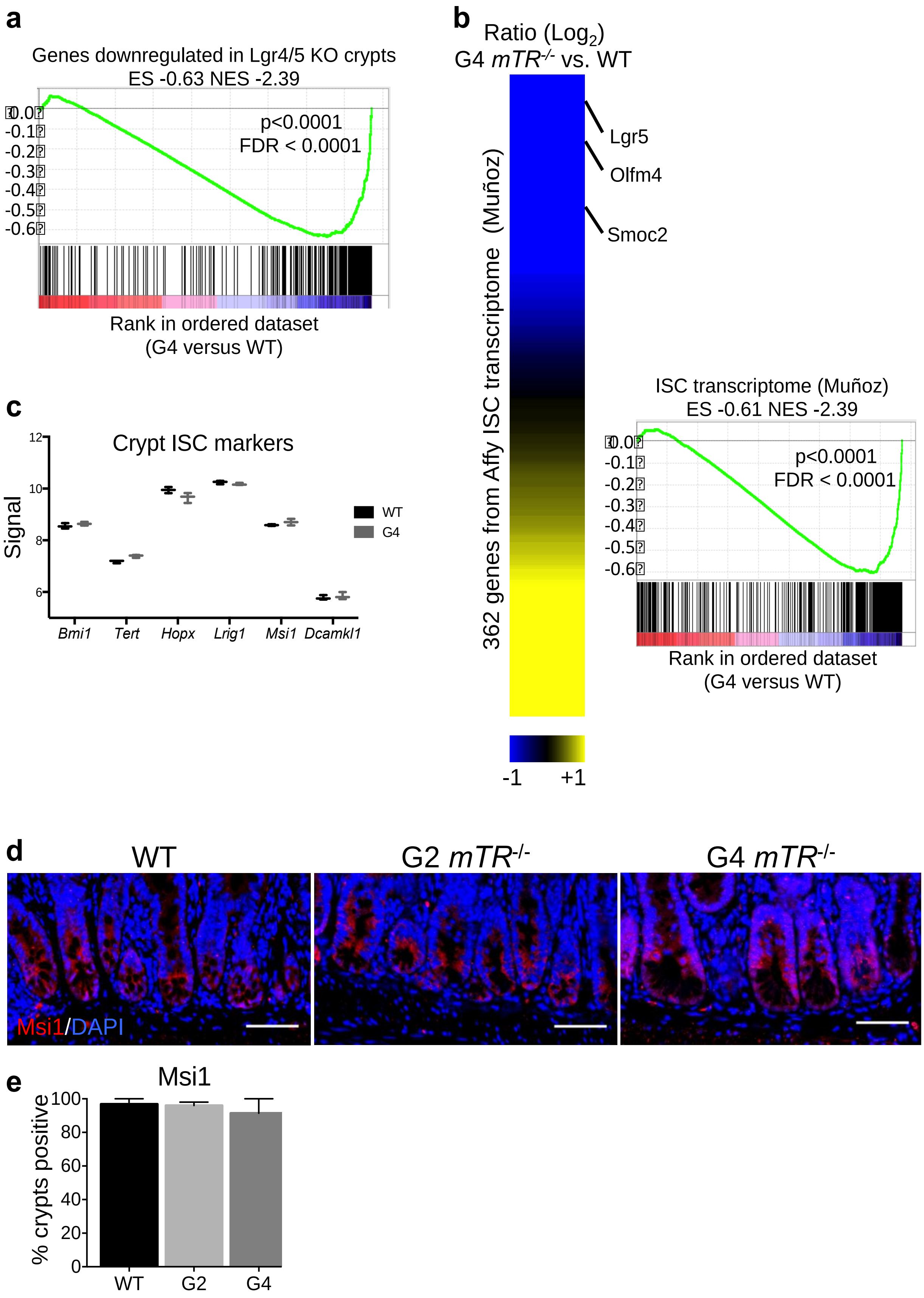
a
Genes downregulated in Lgr4/5 KO crypts
ES -0.63 NES -2.39
0.0
-0.1
-0.2
-0.3
-0.4
-0.5
-0.6
p<0.0001
FDR < 0.0001
Rank in ordered dataset
(G4 versus WT)
b
Ratio (Log2)
G4 mTR-/- vs. WT
Lgr5
Olfm4
Smoc2
362 genes from Affy ISC transcriptome (Muñoz)
ISC transcriptome (Muñoz)
ES -0.61 NES -2.39
0.0
-0.1
-0.2
-0.3
-0.4
-0.5
-0.6
p<0.0001
FDR < 0.0001
Rank in ordered dataset
(G4 versus WT)
-1
+1
c
Crypt ISC markers
Signal
12
10
8
6
WT
G4
Bmi1
Tert
Hopx
Lrig1
Msi1
Dcamkl1
d
WT
G2 mTR-/-
G4 mTR-/-
Msi1/DAPI
e
Msi1
% crypts positive
100
80
60
40
20
0
WT
G2
G4

Supplementary Figure 2. Wnt-dependent, but not Wnt-independent, ISC marker gene transcript levels are unchanged in intestinal crypts of *mTR*$^{-/-}$ mice.

(**a**) Gene Set Enrichment Analysis (GSEA) revealed G4 crypts to have significant downregulation of genes also downregulated in combined *Lgr4* and *Lgr5* KO mouse crypts, which lack Rspo1-responsive Wnt co-receptor proteins that would otherwise ensure robust Wnt signaling[1]. (**b**) GSEA analysis revealed G4 *mTR*$^{-/-}$ ileal crypts to have significantly reduced expression of genes of the ISC transcriptome[2, 3]. (**c**) Array signals of Wnt-independent ISC markers from mRNA expression profiling of isolated WT and G4 *mTR*$^{-/-}$ crypts using Affymetrix Mouse Gene 1.0ST microarrays (*n* = 3). (**d**) WT, G2 and G4 *mTR*$^{-/-}$ crypts are stained for Msi1 protein expression, and (**e**) quantified. Msi1 is an ISC marker that is expressed in a Wnt-independent fashion in +4 LRCs and that persists into CBCs[4, 5], and thus maintenance of Msi1 expression is consistent with CBC-like cell survival (*n* = 3). Scale bars: 50 μm, and error bars are SEMs.

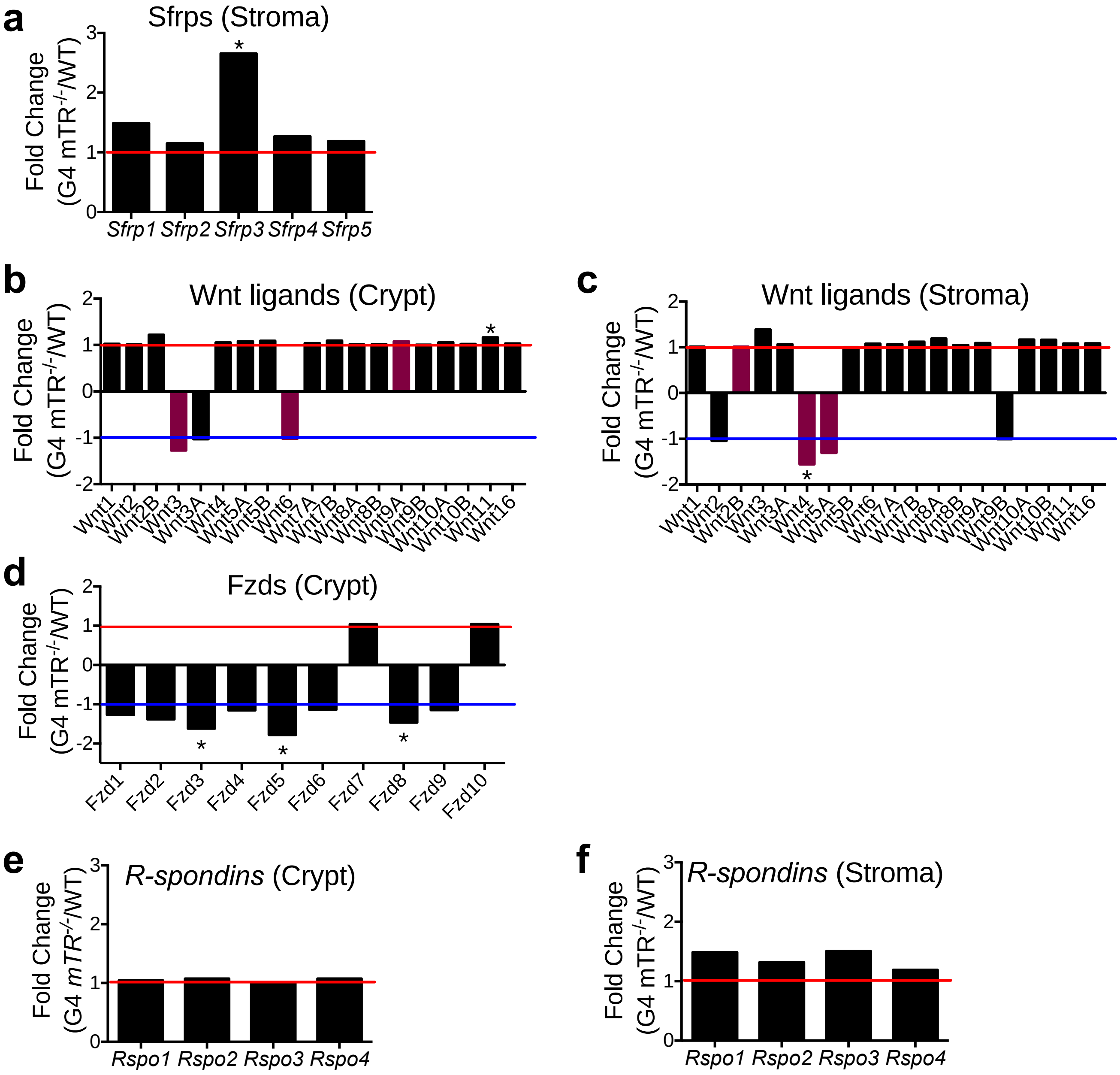
a
Sfrps (Stroma)
Fold Change
(G4 mTR-/-/WT)
3
2
1
0
*
Sfrp1 Sfrp2 Sfrp3 Sfrp4 Sfrp5
b
Wnt ligands (Crypt)
Fold Change
(G4 mTR-/-/WT)
2
1
0
-1
-2
*
Wnt1 Wnt2 Wnt2B Wnt3 Wnt3A Wnt4 Wnt5A Wnt5B Wnt6 Wnt7A Wnt7B Wnt8A Wnt8B Wnt9A Wnt9B Wnt10A Wnt10B Wnt11 Wnt16
c
Wnt ligands (Stroma)
Fold Change
(G4 mTR-/-/WT)
2
1
0
-1
-2
*
Wnt1 Wnt2 Wnt2B Wnt3 Wnt3A Wnt4 Wnt5A Wnt5B Wnt6 Wnt7A Wnt7B Wnt8A Wnt8B Wnt9A Wnt9B Wnt10A Wnt10B Wnt11 Wnt16
d
Fzds (Crypt)
Fold Change
(G4 mTR-/-/WT)
2
1
0
-1
-2
*
*
*
Fzd1 Fzd2 Fzd3 Fzd4 Fzd5 Fzd6 Fzd7 Fzd8 Fzd9 Fzd10
e
R-spondins (Crypt)
Fold Change
(G4 mTR-/-/WT)
3
2
1
0
Rspo1 Rspo2 Rspo3 Rspo4
f
R-spondins (Stroma)
Fold Change
(G4 mTR-/-/WT)
3
2
1
0
Rspo1 Rspo2 Rspo3 Rspo4

Supplementary Figure 3. Microarray gene profiling of the small intestinal stroma and epithelium of WT and G4 *mTR*$^{-/-}$ crypts. (**a-d**) Affymetrix Mouse Gene 1.0ST microarray analyses of mRNA levels were performed on crypts or stroma each isolated from wild-type and G4 *mTR*$^{-/-}$ mice. Examples indicating downregulation of multiple Wnt pathway genes (i.e. Wnt ligands and receptors) and upregulation of Wnt ligand inhibitors (i.e. stromal SFRPs) in G4 *mTR*$^{-/-}$ intestine, which support the GSEA analyses (see Figure 1 and Supplementary Figure 2). (**a**) Fold changes of SFRPs (Wnt ligand inhibitors) in G4 *mTR*$^{-/-}$ intestinal stroma. (**b**) Fold changes of Wnt ligands in G4 *mTR*$^{-/-}$ intestinal crypts and (**c**) stroma; Wnt ligands known to be expressed in robust amounts in each compartment are highlighted in *purple*. (**d**) Fold changes of *Fzd* genes (Wnt ligand receptors) in G4 *mTR*$^{-/-}$ intestinal crypts. (**e**) Fold changes of R-spondins in G4 *mTR*$^{-/-}$ intestinal crypts and (**f**) stroma; * $p<0.05$, based on Benjamini-Hochberg correction of raw p-values.

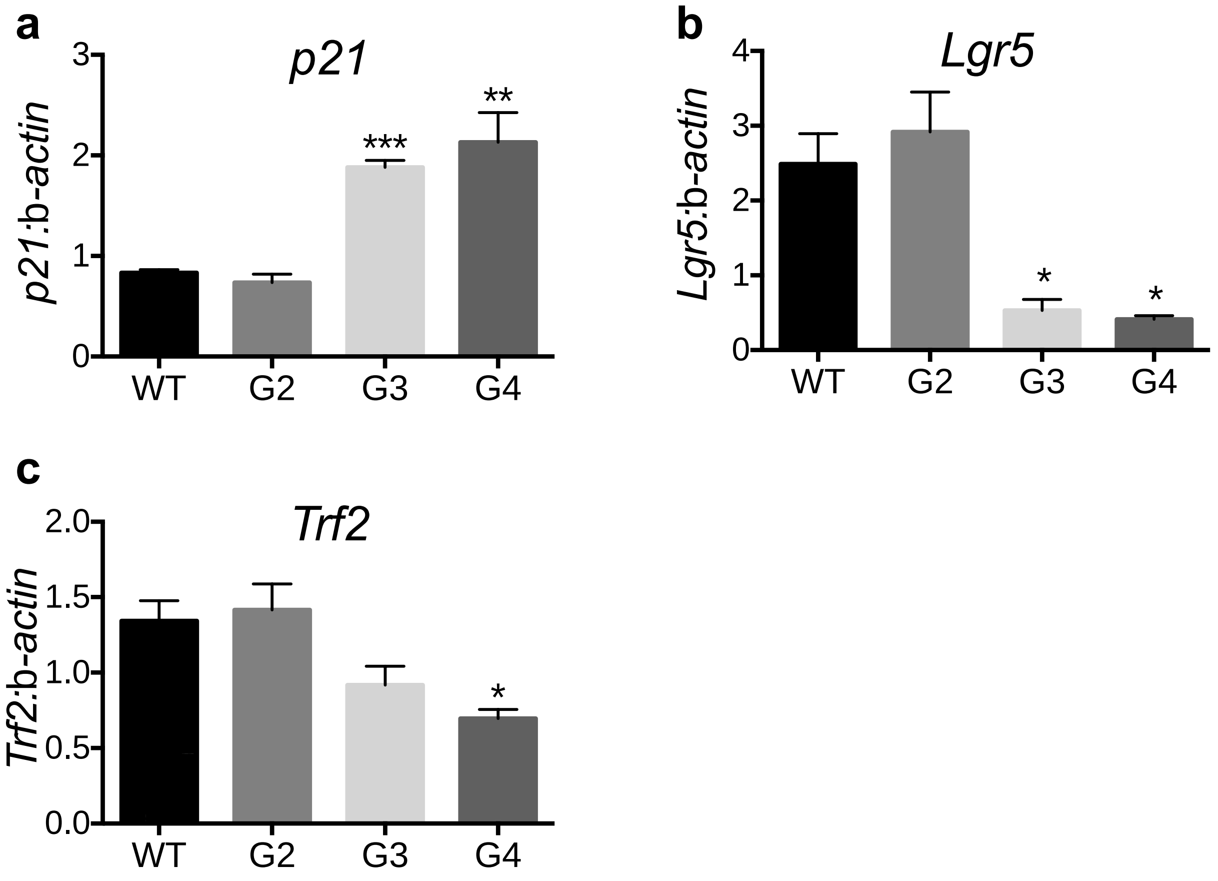
a
p21
p21:b-actin
0
1
2
3
***
**
WT
G2
G3
G4
b
Lgr5
Lgr5:b-actin
0
1
2
3
4
*
*
WT
G2
G3
G4
c
Trf2
Trf2:b-actin
0.0
0.5
1.0
1.5
2.0
*
WT
G2
G3
G4

Supplementary Figure 4. Expression of p53 activated genes, Wnt target genes, and the Trf2 shelterin gene in WT and G4 *mTR*$^{-/-}$ crypts. (**a-c**) qRT-PCR of *p21, Lgr5*, and *Trf2* in WT, G2, G3, and G4 *mTR*$^{-/-}$ crypts (*n* = 4 for WT, G2, G3; *n* = 7 for G4); * $p<0.05$, ** $p<0.005$, *** $p<0.0005$. All error bars reflect standard error of the mean (SEM), and p-values reflect unpaired two-tailed Student's t-tests.

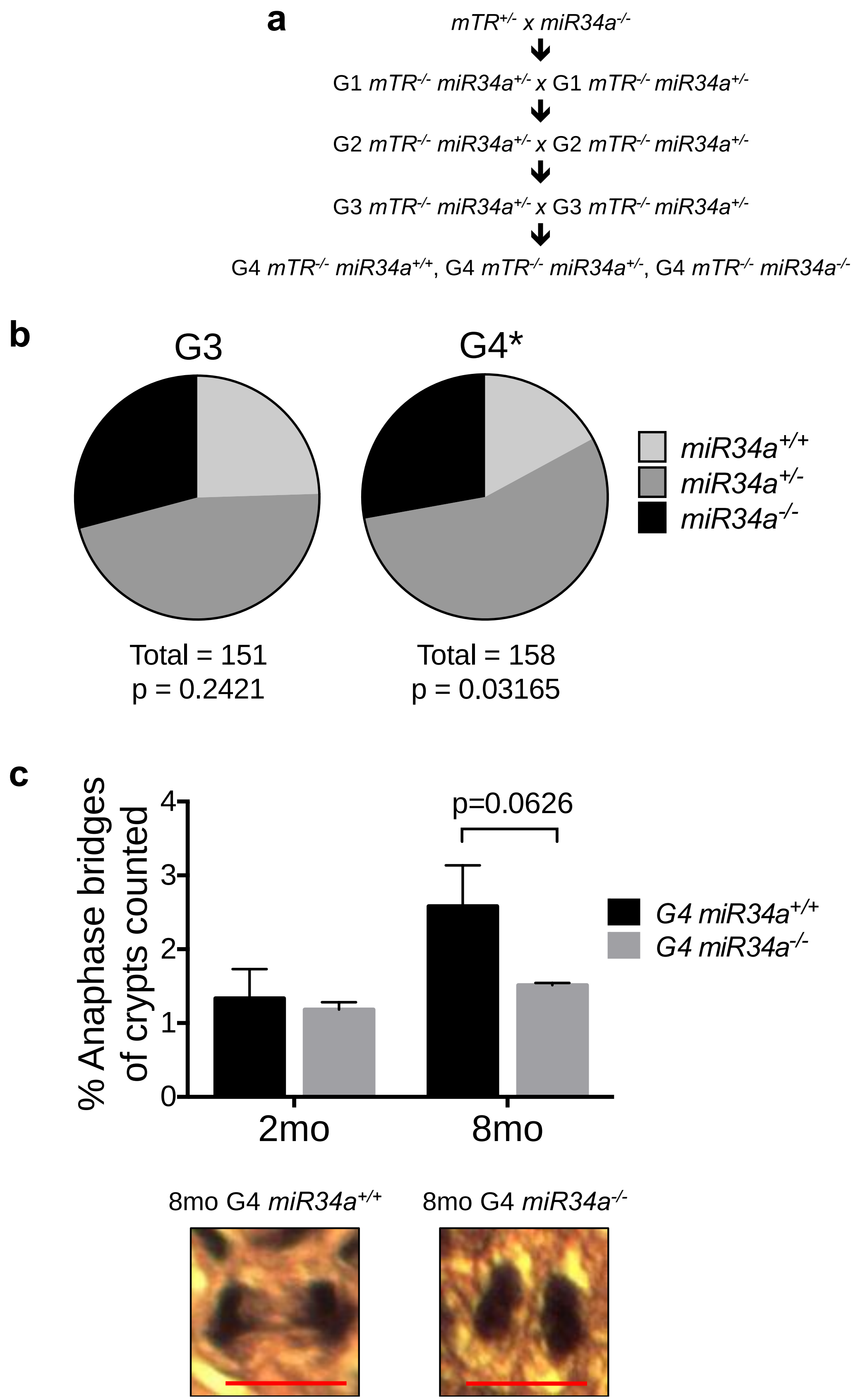
a
mTR+/- x miR34a-/-
G1 mTR-/- miR34a+/- x G1 mTR-/- miR34a+/-
G2 mTR-/- miR34a+/- x G2 mTR-/- miR34a+/-
G3 mTR-/- miR34a+/- x G3 mTR-/- miR34a+/-
G4 mTR-/- miR34a+/+, G4 mTR-/- miR34a+/-, G4 mTR-/- miR34a-/-
b
G3
G4*
miR34a+/+
miR34a+/-
miR34a-/-
Total = 151
p = 0.2421
Total = 158
p = 0.03165
c
% Anaphase bridges of crypts counted
0
1
2
3
4
p=0.0626
G4 miR34a+/+
G4 miR34a-/-
2mo
8mo
8mo G4 miR34a+/+
8mo G4 miR34a-/-

Supplementary Figure 5. (**a**) Breeding scheme for generating *miR34a*$^{-/-}$ onto different generations of *mTR*$^{-/-}$ mice. (**b**) The expected Mendelian frequencies of *miR34a*$^{+/+}$, *miR34a*$^{+/-}$, *miR34a*$^{-/-}$ at G2-G4 are 25%, 50%, and 25%, respectively. The actual birth frequencies of *miR34a*$^{+/+}$, *miR34a*$^{+/-}$, *miR34a*$^{-/-}$ at G3 (25%, 46%, and 29%, respectively) are not significantly different from the expected Mendelian ratios; Chi-squared test one-tailed p = 0.2421 for G3 cohort. At G4, there are significantly more pups born without both copies of *miR34a* than pups with both copies of *miR34a* (i.e. 28% G4 *miR34a*$^{-/-}$ vs 17% G4 *miR34a*$^{+/+}$), suggesting an embryonic/neonatal survival advantage only at later generations of *mTR*$^{-/-}$, when telomeres become critically short and when p53 and miR34a activation would occur; Chi-squared test one-tailed p = 0.03165. (**c**) Similarly, there is a reduction in telomere dysfunction as measured by anaphase bridges that is seen in older (8 month-old) G4 *mTR*$^{-/-}$ *miR34a*$^{-/-}$ mice that is not seen in comparisons in younger (2 month-old) mice; p = 0.0626. Scale bars: 10 μm. All error bars reflect standard error of the mean (SEM), and p-values reflect unpaired two-tailed Student’s t-tests.

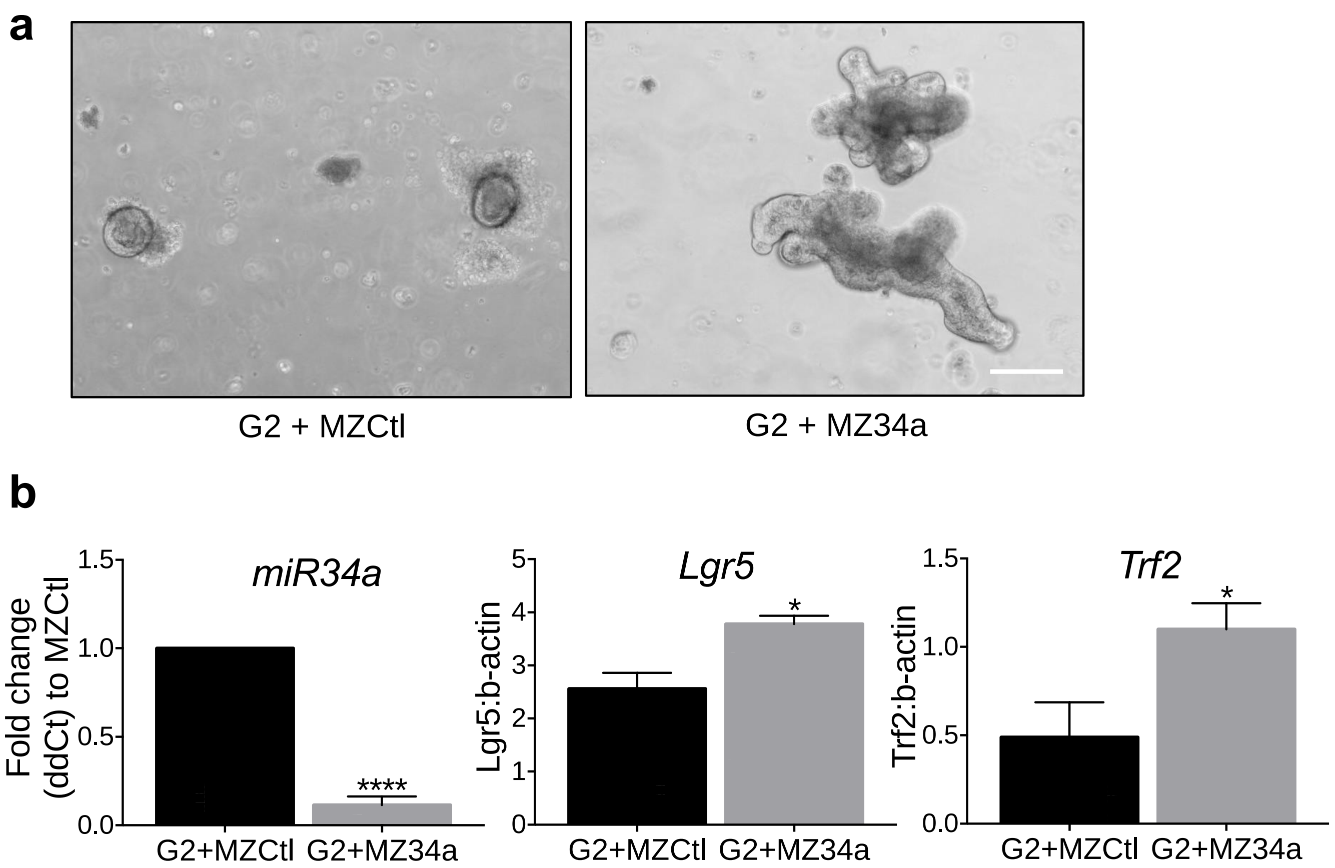
a
G2 + MZCtl
G2 + MZ34a
b
miR34a
Fold change (ddCt) to MZCtl
1.5
1.0
0.5
0.0
****
G2+MZCtl
G2+MZ34a
Lgr5
Lgr5:b-actin
5
4
3
2
1
0
*
G2+MZCtl
G2+MZ34a
Trf2
Trf2:b-actin
1.5
1.0
0.5
0.0
*
G2+MZCtl
G2+MZ34a

Supplementary Figure 6. (**a**) Representative images of G2 *mTR*$^{-/-}$ organoids after puromycin selection after infection with lentivirus expressing anti-scramble microRNA (MZCtl, miRZip-Ctl from Systems Biosciences) or anti-miR34a microRNAs (MZ34a). Note that even though G2 mice had minimal defects in vivo, defects were apparent in organoid cultures. Scale bars: 100 μm. (**b**) qRT-PCR analyses of gene expression for miR34a, Lgr5, Trf2 in G2 *mTR*$^{-/-}$ organoids with MZCtl or MZ34a show rescue of Lgr5 and Trf2 expression after suppression of miR34a. * $p<0.05$, *** $p<0.0005$. All error bars reflect standard error of the mean (SEM), and p-values reflect unpaired two-tailed Student’s t-tests.

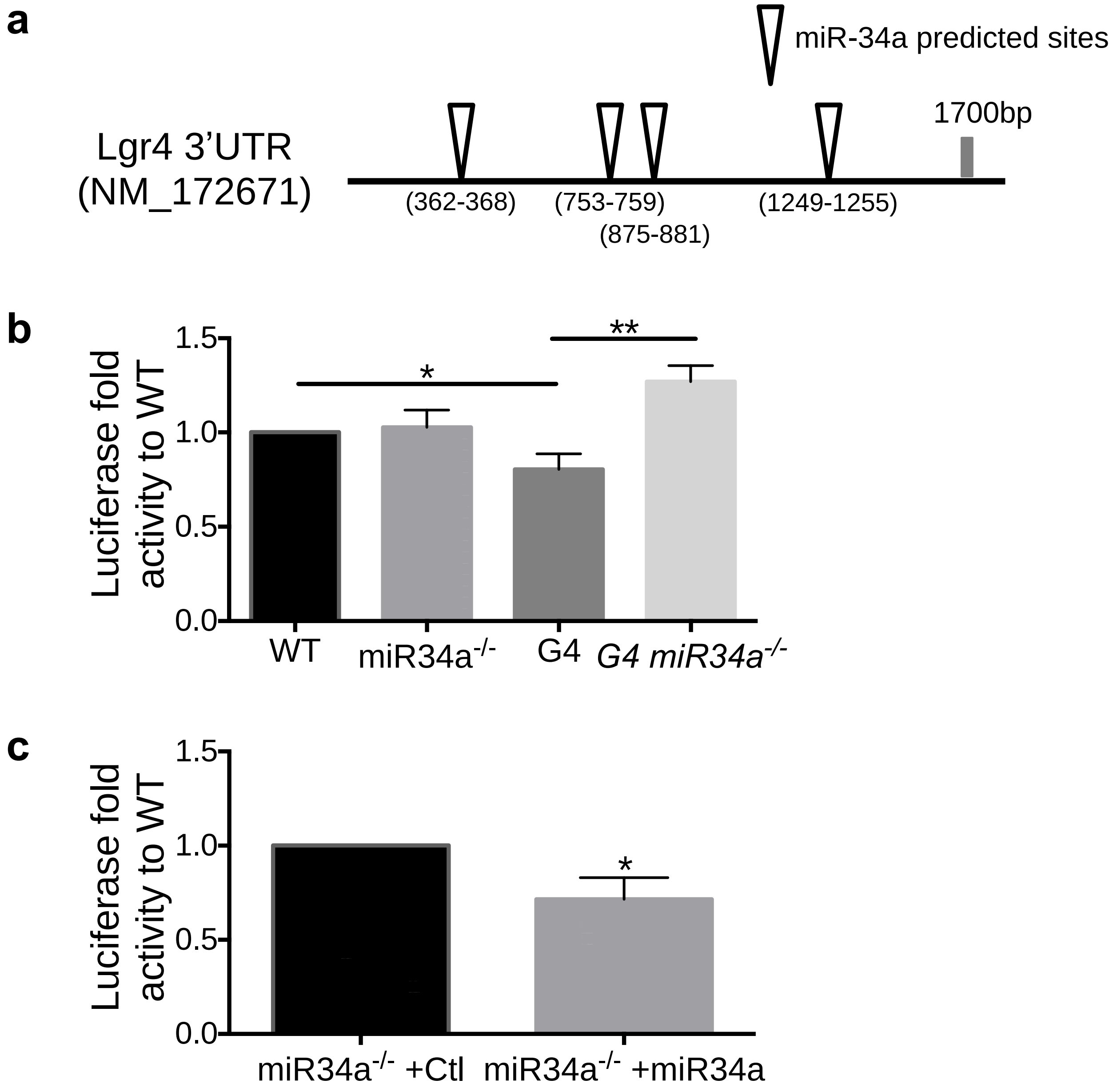
a
miR-34a predicted sites
Lgr4 3'UTR
(NM_172671)
1700bp
(362-368)
(753-759)
(875-881)
(1249-1255)
b
Luciferase fold activity to WT
1.5
1.0
0.5
0.0
*
**
WT
miR34a-/-
G4
G4 miR34a-/-
c
Luciferase fold activity to WT
1.5
1.0
0.5
0.0
*
miR34a-/- +Ctl
miR34a-/- +miR34a

Supplementary Figure 7. (**a**) Predicted miR34a target sites along the 3' UTR of *Lgr4* are indicated by arrowheads. (**b, c**) Reporter constructs with 3'UTR of *Lgr4* cloned downstream of renilla luciferase were transfected into WT, *miR34a*$^{-/-}$, G4 *mTR*$^{-/-}$, or G4 *mTR*$^{-/-}$ *miR34a*$^{-/-}$ mouse skin fibroblasts (**b**), or *miR34a*$^{-/-}$ mouse skin fibroblasts infected with retrovirus overexpressing miR34a or control retrovirus (**c**). Luciferase activity normalized to WT fibroblasts are shown. (n = 7 for WT, *miR34a*$^{-/-}$, G4 *mTR*$^{-/-}$, or G4 *mTR*$^{-/-}$ *miR34a*$^{-/-}$ fibroblasts and n = 4 for infected *miR34a*$^{-/-}$ fibroblasts); * $p<0.05$, ** $p<0.005$. All error bars reflect standard error of the mean (SEM), and p-values reflect unpaired two-tailed Student's t-tests.

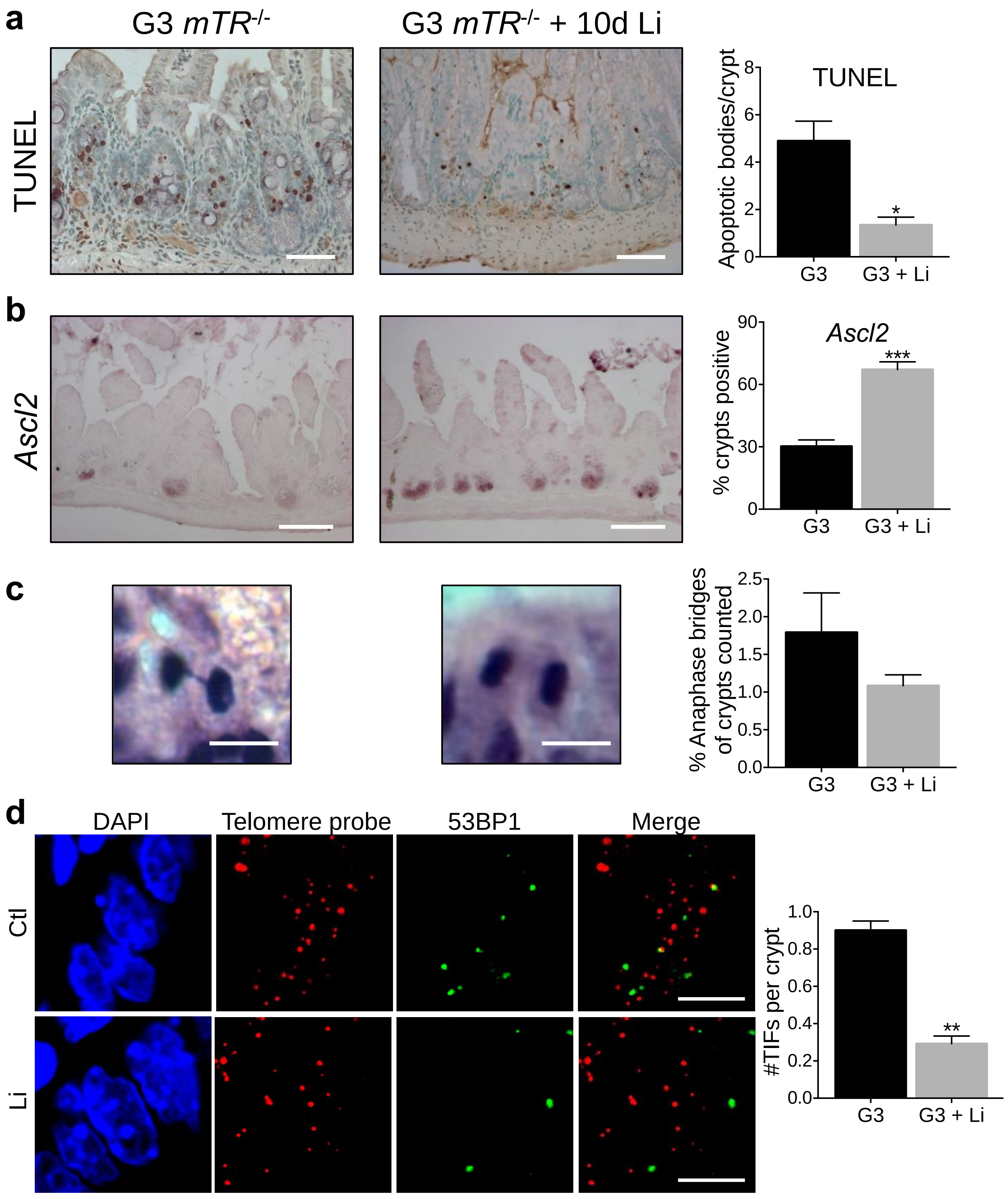

a
G3 mTR-/-
G3 mTR-/- + 10d Li
TUNEL
Apoptotic bodies/crypt
0
2
4
6
8
*
G3
G3 + Li
b
Ascl2
% crypts positive
0
30
60
90
***
c
% Anaphase bridges of crypts counted
0.0
0.5
1.0
1.5
2.0
2.5
d
DAPI
Telomere probe
53BP1
Merge
Ctl
Li
#TIFs per crypt
0.0
0.2
0.4
0.6
0.8
1.0
**

Supplementary Figure 8. Short-term (10-day) lithium treatment rescues G3 *mTR*$^{-/-}$ small intestinal crypt dysfunction and telomere uncapping *in vivo.* (**a**) Representative images showing apoptotic changes by TUNEL-staining of ileum of G3 *mTR*$^{-/-}$ mice treated with the GSK-3 inhibitor lithium in dietary chow (0.212% for 3 days followed by 0.4% for 7 days) and quantitation of TUNEL-positive apoptotic bodies per crypt (right) (*n* = 3 each treated and littermate controls). Scale bars: 50 µm. (**b**) Representative images of *in situ* hybridization for *Ascl2* transcripts in crypts from G3 *mTR*$^{-/-}$ mice treated with Li and quantitation of crypts positive with staining (right) (*n* = 3). Scale bars: 100 µm. (**c**) Representative images showing reduced telomere dysfunction as measured by anaphase bridges (*n* = 3); p=0.1320. Scale bars: 10 µm. (**d**) Representative images showing reduced telomere dysfunction as measured by telomere-dysfunction induced foci (TIFs) in G3 *mTR*$^{-/-}$ mice treated with Li (*n* = 3). Scale bars: 5 µm. Quantitation of anaphase bridges and TIFs (right). For all panels, * p<0.05, ** p<0.005, *** p<0.0005. All error bars reflect standard error of the mean (SEM), and p-values reflect unpaired two-tailed Student’s t-tests.

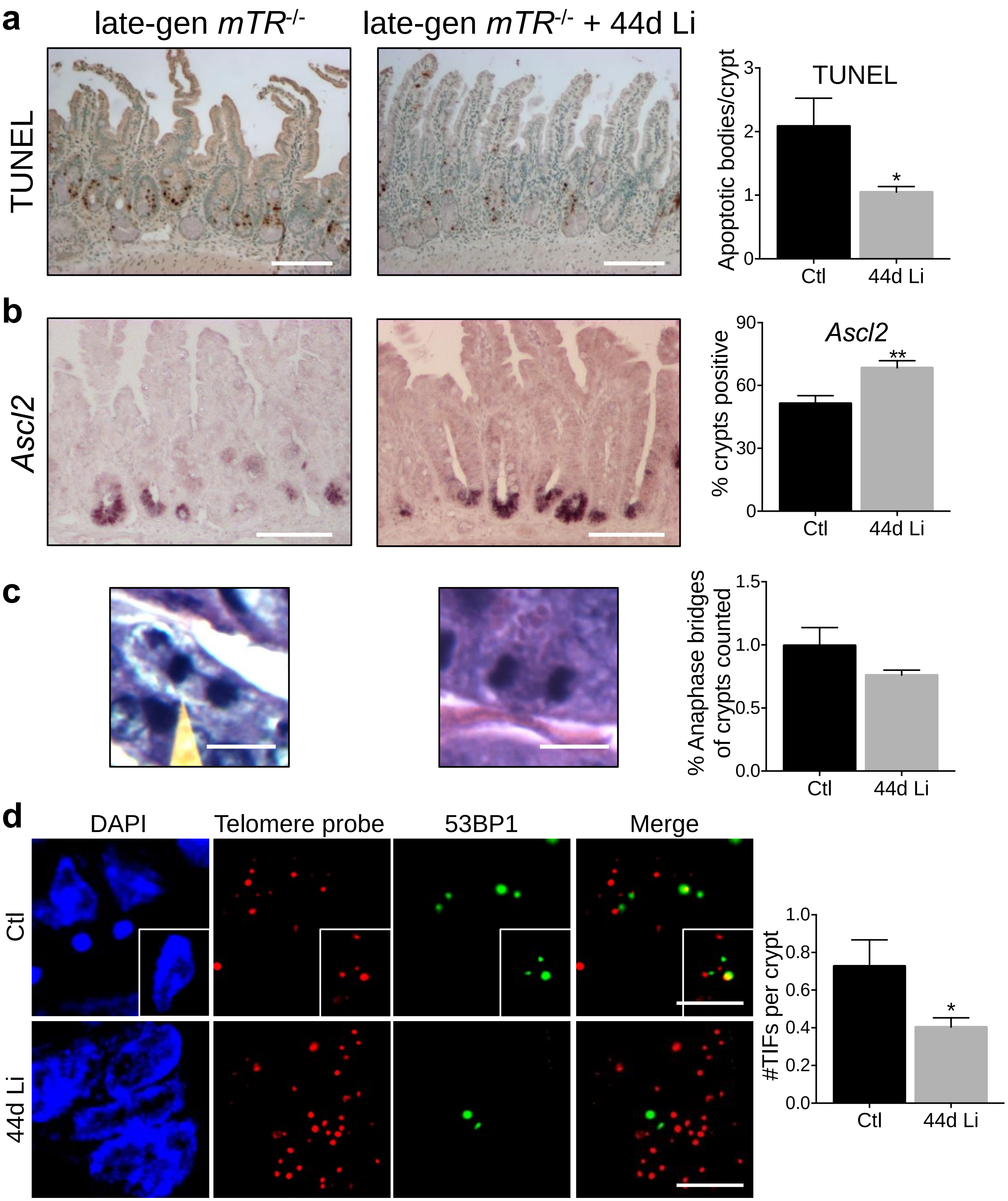

a
late-gen mTR-/-
late-gen mTR-/- + 44d Li
TUNEL
TUNEL
Apoptotic bodies/crypt
0
1
2
3
*
Ctl
44d Li
b
Ascl2
Ascl2
% crypts positive
0
30
60
90
**
Ctl
44d Li
c
% Anaphase bridges of crypts counted
0.0
0.5
1.0
1.5
Ctl
44d Li
d
DAPI
Telomere probe
53BP1
Merge
Ctl
44d Li
#TIFs per crypt
0.0
0.2
0.4
0.6
0.8
1.0
*
Ctl
44d Li

Supplementary Figure 9. Long-term (44-day) lithium treatment rescues late-generation *mTR*$^{-/-}$ small intestinal crypt dysfunction and telomere uncapping *in vivo.* (**a**) Representative images showing apoptotic changes by TUNEL-staining of ileum of late-generation *mTR*$^{-/-}$ mice treated with the GSK-3 inhibitor lithium in dietary chow (4 consecutive cycles of 0.212% for 4 days followed by 0.4% for 7 days) and quantitation of TUNEL-positive apoptotic bodies per crypt (right) (*n* = 4 each treated and littermate controls). Scale bars: 100 μm. (**b**) Representative images of *in situ* hybridization for *Ascl2* transcripts in crypts from late-generation *mTR*$^{-/-}$ mice treated with Li and quantitation of crypts positive with staining (right) (*n* = 4). Scale bars: 100 μm. (**c**) Representative images showing reduced telomere dysfunction as measured by anaphase bridges (*n* = 4); p=0.0704. Scale bars: 10 μm (**d**) and telomere-dysfunction induced foci (TIFs) in late-generation *mTR*$^{-/-}$ mice treated with Li (*n* = 4). Scale bars: 5 μm. Quantitation of anaphase bridges and TIFs (right). For all panels, * p<0.05, ** p<0.005. * p<0.05, ** p<0.005, *** p<0.0005. All error bars reflect standard error of the mean (SEM), and p-values reflect unpaired two-tailed Student's t-tests. Also of note, no cancerous tumors were observed in mice after 44-day treatment with lithium.

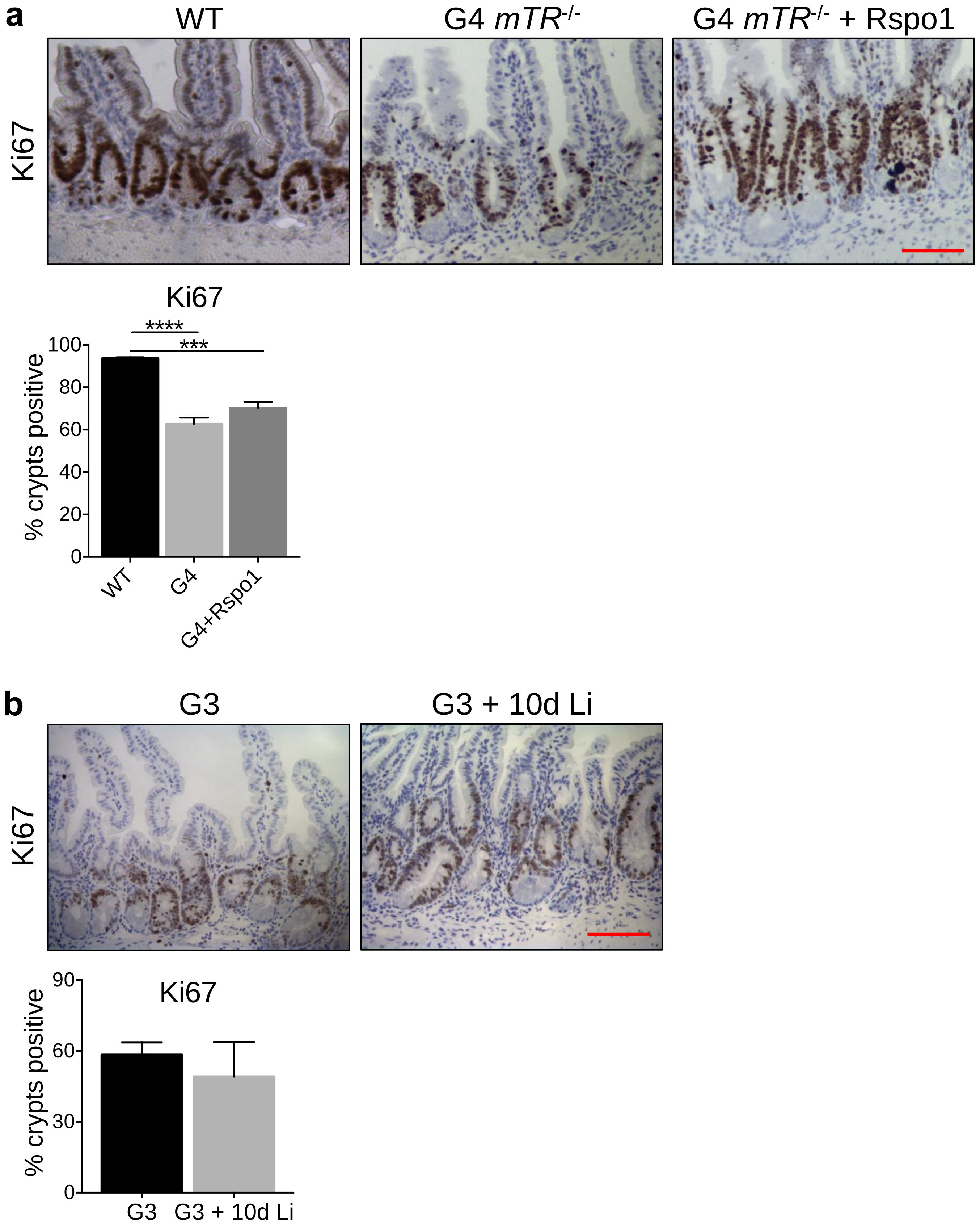

a
WT
G4 mTR-/-
G4 mTR-/- + Rspo1
Ki67
Ki67
****
***
% crypts positive
100
80
60
40
20
0
WT
G4
G4+Rspo1
b
G3
G3 + 10d Li
Ki67
Ki67
% crypts positive
90
60
30
0
G3
G3 + 10d Li

Supplementary Figure 10. Ki67 staining in WT, G4 *mTR*$^{-/-}$, Rspo1-treated G4 *mTR*$^{-/-}$, G3 *mTR*$^{-/-}$, Li-treated G3 *mTR*$^{-/-}$ small intestinal crypts. Representative images of Ki67-stained ileum of WT, G4 *mTR*$^{-/-}$, and Rspo1-treated G4 *mTR*$^{-/-}$ mice (**a**) or G3 *mTR*$^{-/-}$, and Li-treated G3 *mTR*$^{-/-}$ mice (**b**). Scale bars: 50 μm. Although enhanced proliferation was apparent in the transit amplifying regions, consistent with previous findings[6], no significant increase apparent proliferation of cells in crypts was observed. This is not surprising, as the diminished apoptosis afforded by the Wnt pathway agonists would diminish the demand on stem cells to generate new progeny to maintain tissue homeostasis. All error bars reflect standard error of the mean (SEM), and p-values reflect unpaired two-tailed Student's t-tests (*** $p<0.0005$, **** $p<0.0001$).

a

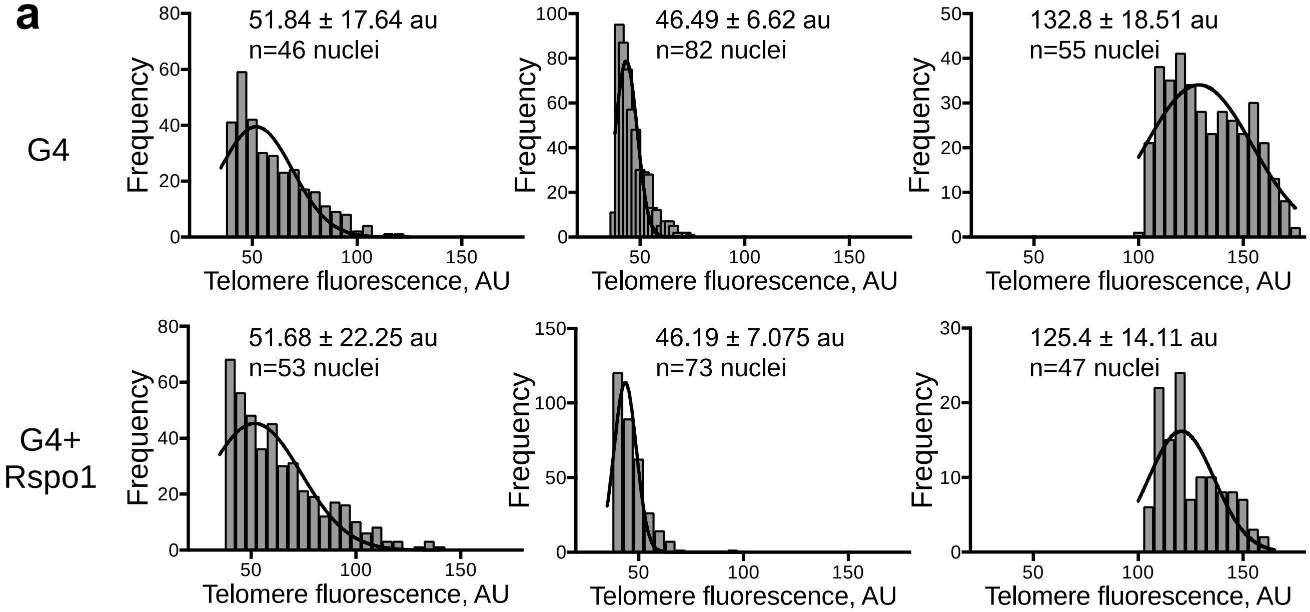

Difference between medians	+3.387 (longer in Rspo1-treated mouse)	-0.28 (shorter in Rspo1-treated mouse)	-8.251 (shorter in Rspo1-treated mouse)
Significant?	No (p=0.103)	No (p=0.269)	Yes (p<0.0002)

b

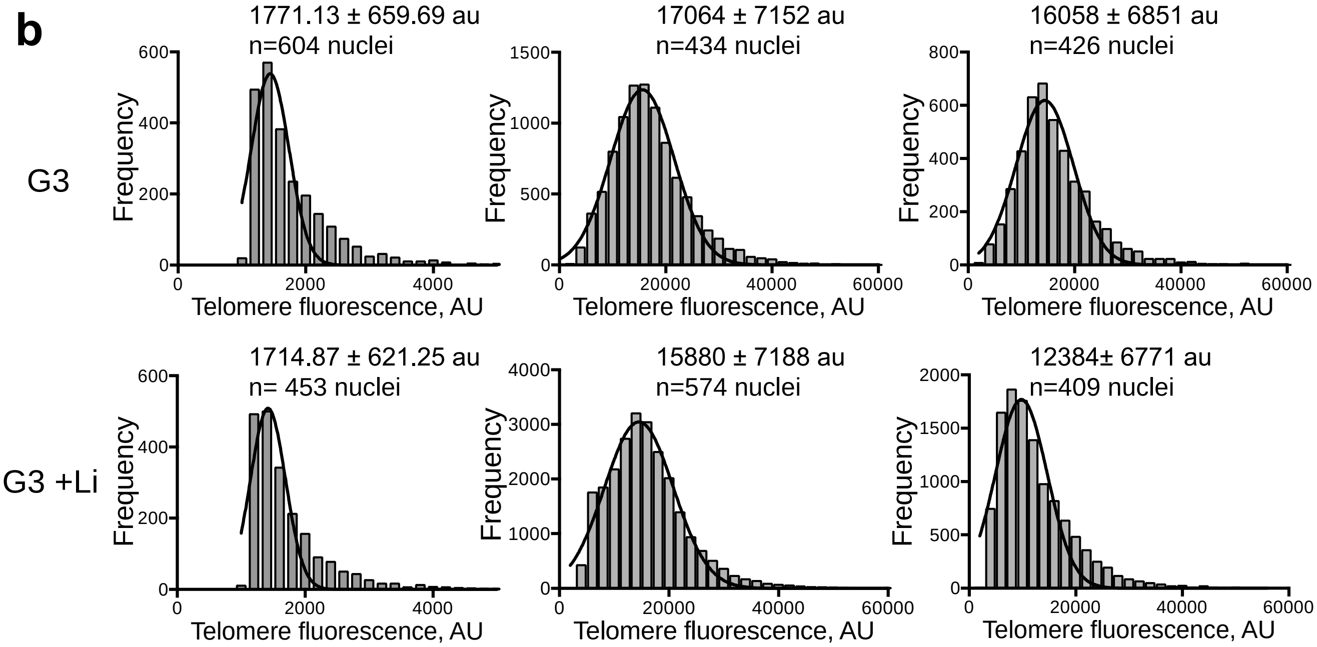

Difference between medians	-33.52 (shorter in Li-treated mouse)	-1071 (shorter in Li-treated mouse)	-4111 (shorter in Li-treated mouse)
Significant?	Yes (p<0.0007)	Yes (p<0.0001)	Yes (p<0.0001)

Supplementary Figure 11. Histograms of telomere fluorescence by Q-FISH analysis of intestinal crypts of G4 *mTR*$^{-/-}$ mice treated with Rspo1 and or G3 *mTR*$^{-/-}$ treated with **lithium-chow.** (**a**) Histograms of telomere fluorescence frequency by Q-FISH from three pairs of littermate control and Rspo1-treated G4 *mTR*$^{-/-}$ mouse intestinal sections, hybridized with Cy3 telomere-PNA probes. All comparisons were made on tissues imaged on the same day, and because intensities varied between different days, the comparisons were not averaged. (**b**) Histograms of telomere fluorescence frequency by Q-FISH from three pairs of control and Li-treated G3 *mTR*$^{-/-}$ mouse intestinal sections, hybridized with Cy3 telomere-PNA probes and imaged on different days; hence comparisons were not averaged. Mean and SD of telomere fluorescence, and the number of nuclei analyzed, are indicated on each graph. Differences in median telomere lengths between control and Rspo1 or Li-treated mice were determined by the Mann-Whitney U-test.

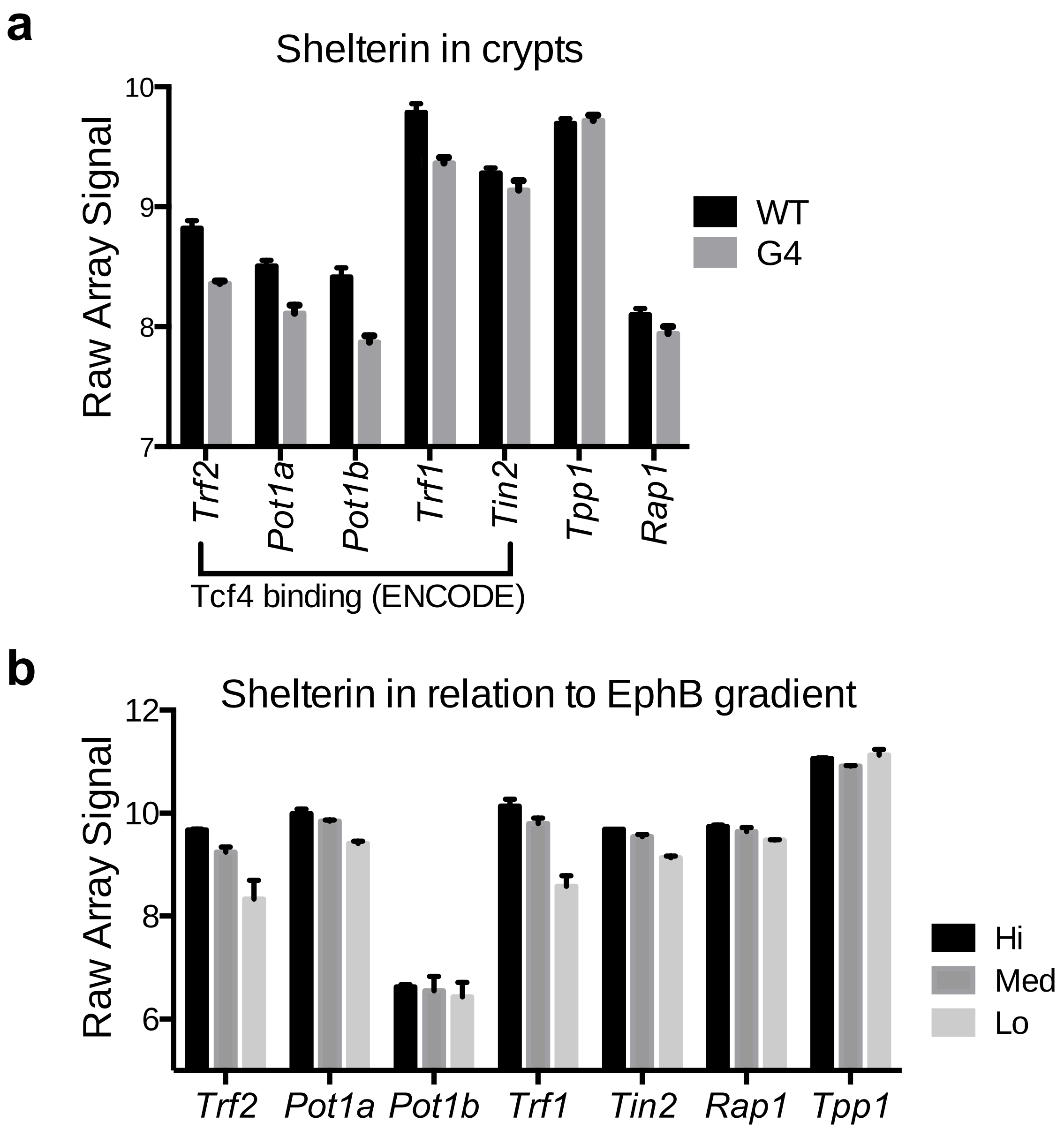

a
Shelterin in crypts
Raw Array Signal
10
9
8
7
WT
G4
Trf2
Pot1a
Pot1b
Trf1
Tin2
Tpp1
Rap1
Tcf4 binding (ENCODE)
b
Shelterin in relation to EphB gradient
Raw Array Signal
12
10
8
6
Hi
Med
Lo
Trf2
Pot1a
Pot1b
Trf1
Tin2
Rap1
Tpp1

Supplementary Figure 12. Expression of shelterin genes in WT vs G4 *mTR*$^{-/-}$ crypts, and distribution of shelterin gene expression in crypt cells expressing different levels of EphB2. (**a**) Microarray gene profiling of shelterin genes in WT and G4 *mTR*$^{-/-}$ crypts. (**b**) Microarray gene profiling of shelterin genes in crypt epithelial cells sorting according to surface expression levels of EphB2 (*high, medium* or *low*)[7]. *EphB2* is a Wnt target gene that is expressed in a gradient similar to that of Wnt activity declining upward from the base of the crypt. All values are means (n = 3) and SEMs.

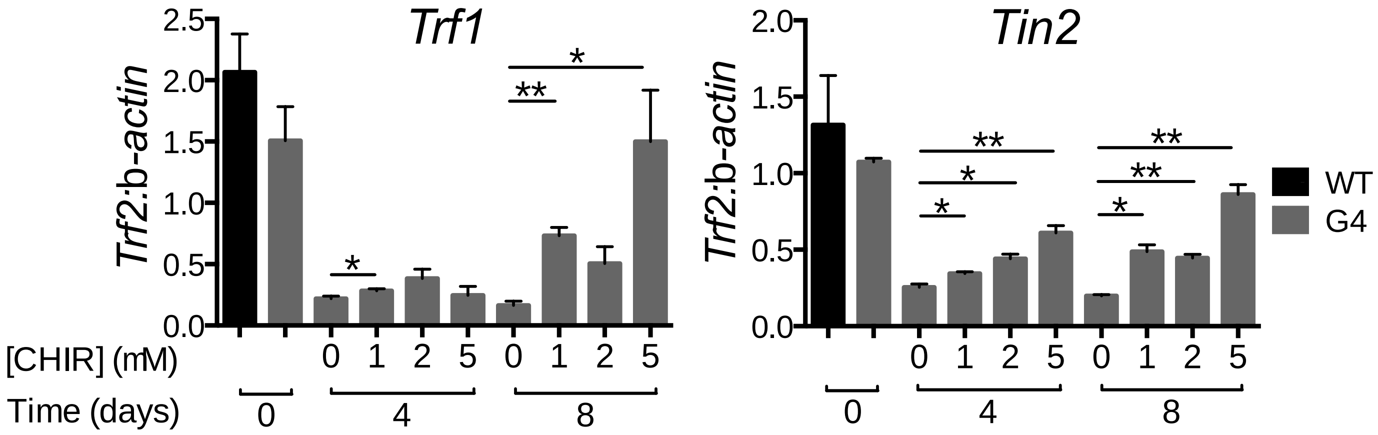

Trf1
Tin2
Trf2:b-actin
2.5
2.0
1.5
1.0
0.5
0.0
WT
G4
[CHIR] (mM)
0 1 2 5 0 1 2 5
Time (days)
0
4
8
*
**

Supplementary Figure 13. *Trf1* and *Tin2* expression in cultured G4 *mTR*$^{-/-}$ intestinal organoids treated with CHIR99021. qRT-PCR measurement of *Trf1* and *Tin2* transcripts in WT and G4 *mTR*$^{-/-}$ crypts at t=0, and treated with increasing doses of CHIR99021 at days 4 or 8 (n = 3); * $p<0.01$, ** $p<0.005$. All error bars reflect standard error of the mean (SEM), and p-values reflect unpaired two-tailed Student's t-tests.

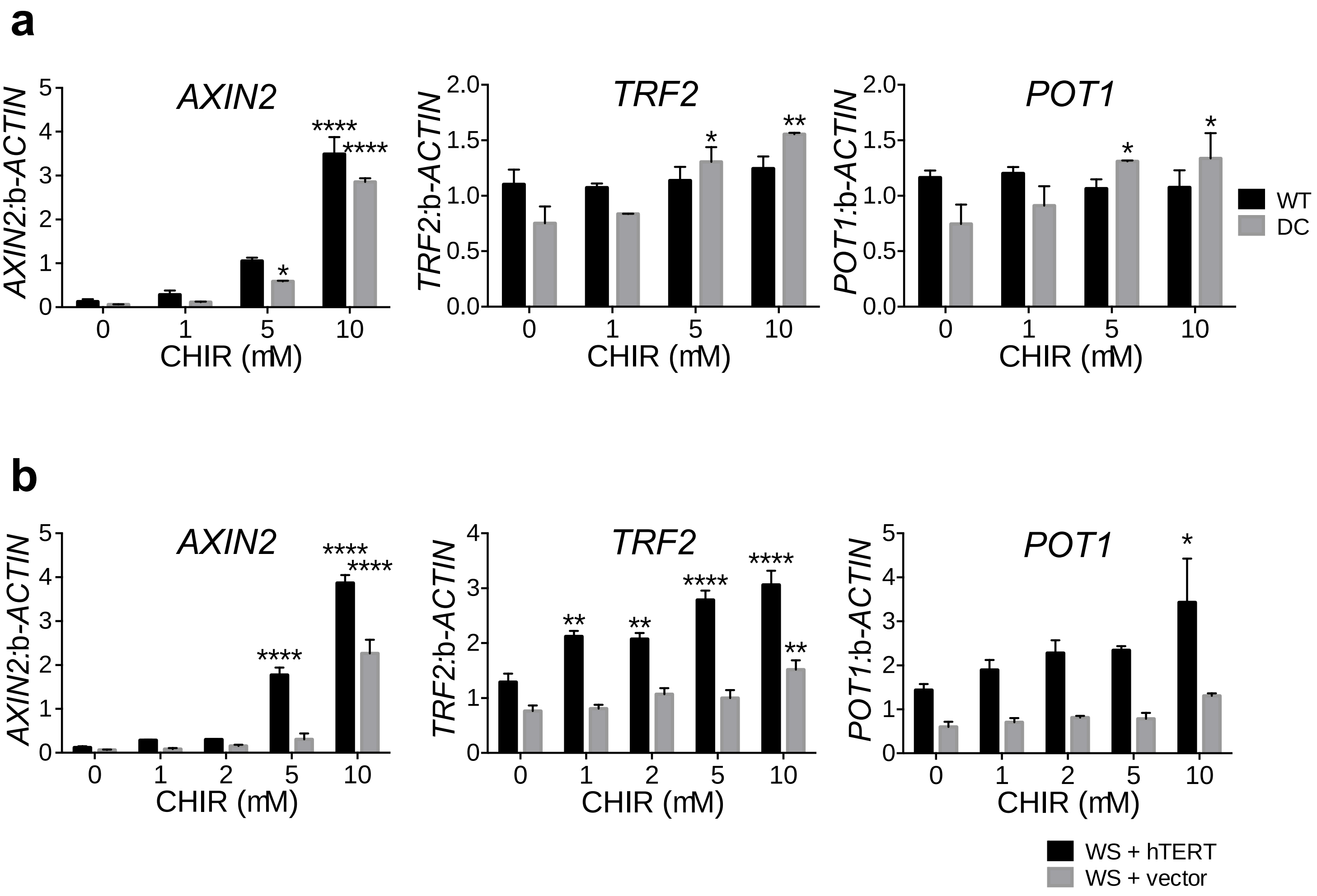
a
AXIN2
AXIN2:b-ACTIN
CHIR (mM)
TRF2
TRF2:b-ACTIN
POT1
POT1:b-ACTIN
WT
DC
b
AXIN2
AXIN2:b-ACTIN
CHIR (mM)
TRF2
TRF2:b-ACTIN
POT1
POT1:b-ACTIN
WS + hTERT
WS + vector

Supplementary Figure 14. Primary human fibroblasts with telomere dysfunction display reduced expression of WNT pathway target genes including shelterins, which is rescued by CHIR99021 or telomere length correction by telomerase overexpression. (**a, b**) Expression of *AXIN2*, *TRF2*, and *POT1* mRNA levels in primary human fibroblasts from (**a**) three healthy people (WT) and two people with dyskeratosis congenita (DC) treated with the indicated levels of CHIR99021 for 48 hours, and (**b**) a person with Werner syndrome (WS) infected with retrovirus expressing hTERT or a vector control and treated with the indicated levels of CHIR99021 for four days; * $p<0.05$, ** $p<0.005$, **** $p<0.0001$. All error bars reflect standard error of the mean (SEM), and p-values reflect a two-way ANOVA. hTERT overexpression is known to rescue numerous defects in WS cells, which otherwise suffer from premature telomere losses[8].

Supplementary Table 1. Top 5 GSEA gene sets downregulated in G4 *mTR*$^{-/-}$ crypts

Gene set name in MSigDB	ES	NES	FDR	Gene set description
ZHANG_TLX_TARGETS_UP	-0.75	-2.41	0.000	Genes upregulated in neural stem cells after cre-lox TLX knockout
**FEVR_CTNNB1_TARGETS_DN**	**-0.58**	**-2.27**	**0.000**	**Genes downregulated in intestinal crypt cells upon β-catenin deletion**
PUJANA_XPRESS_INT_NETWORK	-0.65	-2.36	0.000	Intersection of genes correlating with BRCA1, BRCA2, ATM, and CHEK2 in normal tissues
REN_BOUND_BY_E2F	-0.69	-2.32	0.000	Genes whose promoters were bound by E2F1 and E2F4 by ChIP in WI-38 fibroblasts
PUJANA_BRCA_CENTERED_NETWORK	-0.66	-2.31	0.000	Genes constituting the BRCA-centered network (genes potentially associated with higher breast cancer risk)

Supplementary Table 1. Gene set enrichment analysis (GSEA) of G4 *mTR*$^{-/-}$ versus WT intestinal crypt epithelia. Top 5 gene sets downregulated in G4 *mTR*$^{-/-}$ crypts compared to WT crypts as determined by GSEA, using the C2 (curated) database of gene sets published in the Molecular Signature Database version 3.1.

Transcript ID	Fold-Change	q-value(%)	p-value	Targets experimentally validated/Properly annotated?
mmu-miR-5097	9.6531	0	9.97E-05	misannotation
mmu-miR-3096b-5p	9.59491	0	0.000246684	misannotation
mmu-miR-1983	8.61188	0	0.000155303	dicer processed from alt tRNA; unknown biologic function/significance
mmu-miR-34a	7.51112	0	0.000160584	Yes
mmu-miR-3472	7.47828	0	0.000360328	No
mmu-miR-712-star	6.93032	0	0.000385375	712 form is potential biomarker for atherosclerosis
mmu-miR-3473b	6.24534	0	0.000406614	No
mmu-miR-122	5.96007	0.452051	0.00615764	Yes
mmu-miR-2137	5.33762	0	1.66E-05	No
mmu-miR-3096b-3p	5.31508	0.812547	0.0178771	misannotation
mmu-miR-195-star	5.07189	2.45988	0.0576239	Yes
mmu-miR-5122	4.82135	0	0.000685543	No
mmu-miR-326-star	4.79776	0	0.00231744	Yes
mmu-miR-23a-star	4.79226	0.452051	0.00536474	Yes
mmu-miR-3473	4.57791	0	0.00252394	Multiple forms (a-g) exist
mmu-miR-3093-3p	4.3942	0	0.00120212	No, but high annotation confidence
mmu-miR-3096-3p	4.36136	0.452051	0.0127714	misannotation
mmu-miR-711	4.35402	0	0.00293431	No
mmu-miR-92b-star	4.04567	0	0.00339936	Yes
mmu-miR-5115	3.88034	0.452051	0.0057162	misannotation
mmu-miR-1906	3.87792	0	0.000513075	No
mmu-miR-762	3.86672	0	0.000259164	No
mmu-miR-714	3.58317	0	0.00035751	No
mmu-miR-135a-1-star	3.58272	0	0.00202011	Yes
mmu-miR-16-1-star	3.57839	1.27744	0.0287758	Yes
mmu-miR-1982-star	3.50934	0	0.00268075	No
mmu-miR-712	3.50561	0	0.000685905	712 form is potential biomarker for atherosclerosis
mmu-miR-1894-3p	3.45166	0	0.000701415	No
mmu-miR-1224	3.42053	0	0.00131013	No, but confirmed by extensive cloning
mmu-miR-298	3.33754	0	0.00156887	Yes
mmu-miR-1249-star	3.31616	0.452051	0.00414234	No, but high annotation confidence
mmu-miR-3096-5p	3.26607	0.452051	0.00769501	misannotation
mmu-miR-223	3.2555	2.80972	0.0590441	Yes
mmu-miR-1949	3.21146	0.452051	0.00905799	No
mmu-miR-5130	3.1989	0	4.78E-05	dicer processed from alt tRNA; unknown biologic function/significance
mmu-miR-3104-5p	3.16027	0	0.000953081	No
mmu-miR-5131	3.14965	0.452051	0.00854881	No
mmu-miR-1893	3.10376	0.452051	0.00463347	No
mmu-miR-1945	3.08492	0	0.00107002	No
mmu-miR-134	3.07513	0.452051	0.0123926	Yes
mmu-miR-210	3.05249	1.4998	0.0332925	Yes
mmu-miR-3067-star	3.02973	1.65299	0.0354962	No
mmu-miR-34a-star	2.99366	1.0581	0.019744	Yes
mmu-miR-874	2.99158	0.452051	0.00895706	Yes
mmu-miR-1946b	2.9373	0.452051	0.010274	No

Transcript ID	Fold-Change	q-value(%)	p-value	Targets experimentally validated/Properly annotated?
mmu-miR-1956	2.81825	1.27744	0.029318	No
mmu-miR-5109	2.78966	0	0.000800544	misannotation
mmu-miR-128-2-star	2.75121	3.06646	0.0627308	Yes 128
mmu-miR-150-star	2.71079	0.452051	0.0110623	Yes, 150
mmu-miR-3100-5p	2.68017	0.812547	0.0136969	No
mmu-miR-125a-3p	2.67004	2.45988	0.0460841	Yes, mir-10 family
mmu-miR-669f-5p	2.64649	1.79736	0.0431291	No, but high annotation confidence
mmu-miR-696	2.6414	1.79736	0.0391278	No
mmu-miR-1903	2.61401	0	0.00309135	No
mmu-miR-669m-5p	2.60099	1.0581	0.018985	No, but high annotation confidence
mmu-miR-211-star	2.56976	0	8.79E-05	Yes
mmu-miR-328-star	2.56062	0.452051	0.00564782	Yes
mmu-miR-3077-star	2.53872	0	0.000798008	No
mmu-miR-1247-star	2.48884	0.452051	0.00573571	No, but high annotation confidence
mmu-miR-3102-star	2.47055	0	0.000266576	No
mmu-miR-5128	2.47028	0.452051	0.00437516	No
mmu-miR-323-5p	2.45919	0	0.00222691	No, but belongs to mir-154 family
mmu-miR-199b-star	2.44546	2.45988	0.0468748	Yes
mmu-miR-346-star	2.4203	0.452051	0.00488107	Yes
mmu-miR-760-3p	2.38472	0	0.00117428	No, but high annotation confidence
mmu-miR-24-2-star	2.36329	0.452051	0.00775253	Yes
mmu-miR-504-star	2.36199	4.77456	0.0859973	Yes, -504 form negatively reg p53
mmu-miR-27a	2.35721	0	0.00159966	Yes
mmu-miR-3092	2.34248	0.452051	0.00832069	No
mmu-miR-370	2.33504	2.80972	0.0516444	Yes
mmu-miR-770-3p	2.32274	0.452051	0.00699165	No, but high annotation confidence
mmu-miR-677-star	2.31572	0.812547	0.01714	No
mmu-miR-1943	2.30496	0.452051	0.00323731	No, but high annotation confidence
mmu-miR-615-5p	2.30494	2.80972	0.0503338	Yes
mmu-miR-3090-star	2.29738	0.452051	0.00701325	No
mmu-miR-149-star	2.27879	0	0.00217518	Yes, 149
mmu-miR-3113	2.27667	0.812547	0.0157293	No
mmu-miR-5120	2.2574	0	0.0020514	No
mmu-miR-92a-2-star	2.25607	0.452051	0.0108127	Yes
mmu-miR-23a	2.24186	0	0.00117961	Yes
mmu-miR-668	2.23182	0.452051	0.00986125	No, but high annotation confidence
mmu-miR-1940	2.22367	2.45988	0.0439324	No
mmu-miR-494	2.18796	0.812547	0.0161219	No
mmu-miR-2182	2.15854	1.27744	0.0240374	No
mmu-miR-466j	2.15556	2.45988	0.0472792	No
mmu-miR-193b-star	2.14098	0.452051	0.00325434	Yes
mmu-miR-129-5p	2.12663	2.80972	0.0536045	Yes
mmu-miR-297a	2.09988	1.27744	0.0226491	Yes
mmu-miR-212-3p	2.08676	1.0581	0.0189996	Yes
mmu-miR-214	2.08411	0.452051	0.0073284	Yes

Transcript ID	Fold-Change	q-value(%)	p-value	Targets experimentally validated/Properly annotated?
mmu-miR-1946a	2.05986	0.812547	0.0114048	No
mmu-miR-3087-star	2.05106	0	3.73E-05	No
mmu-miR-22	2.04582	0.452051	0.00520935	Yes
mmu-miR-2136	2.02175	4.77456	0.0739416	No
mmu-miR-5119	1.99957	1.79736	0.0383339	No
mmu-miR-720	1.99698	3.7983	0.0718273	misannotation
mmu-miR-27a-star	1.98932	2.45988	0.0414182	Yes
mmu-miR-718	1.98486	0	0.000167688	No
mmu-miR-2861	1.94264	0	0.00022762	Yes, clustered with 3960
mmu-miR-5126	1.92859	0	0.0011451	No
mmu-miR-466m-5p	1.92267	3.06646	0.0591985	No, but high annotation confidence
mmu-miR-204-star	1.92027	1.79736	0.0367601	Yes
mmu-miR-1199	1.91628	3.06646	0.0540421	No
mmu-miR-3960	1.89882	0	0.000164254	Yes
mmu-miR-705	1.88755	0.452051	0.00760694	No
mmu-miR-1892	1.88512	1.0581	0.015258	No
mmu-miR-3091-5p	1.88396	0	0.0009681	No, but high annotation confidence
mmu-miR-497	1.88045	3.06646	0.0563177	Yes
mmu-miR-678	1.87363	0.452051	0.00420737	No
mmu-miR-29b-2-star	1.87293	2.45988	0.039491	Yes
mmu-miR-5111	1.85493	1.4998	0.0250187	misannotation
mmu-miR-132	1.85284	0.452051	0.00720396	Yes
mmu-miR-5105	1.85202	0.452051	0.00359015	misannotation
mmu-miR-1934-star	1.82831	0.452051	0.0041777	No, but high annotation confidence
mmu-miR-667	1.81784	2.80972	0.0464517	No, but high annotation confidence
mmu-miR-365-2-star	1.81141	0.452051	0.00637733	Yes
mmu-miR-290-5p	1.80764	3.7983	0.0639913	Yes

Transcript ID	Fold-Change	q-value(%)	p-value	Targets experimentally validated/Properly annotated?
mmu-miR-363-5p	-6.78023	0	0.000189718	Yes
mmu-miR-363-3p	-6.62343	0.844622	0.00418848	Yes
mmu-miR-181c	-5.84167	0	0.000915913	Yes
mmu-miR-150	-5.24663	2.80972	0.0363501	Yes
mmu-miR-181b	-4.52732	0	4.12E-05	Yes
mmu-miR-181a	-4.19944	0	0.000159743	Yes
mmu-miR-10a-star	-3.95851	0	0.00194124	Yes, mir-10 family
mmu-miR-7a-1-star	-3.47662	1.08799	0.0116818	Yes
mmu-miR-181c-star	-3.29686	0.844622	0.00246322	Yes
mmu-miR-196a-1-star	-3.29128	2.45988	0.0296813	Yes
mmu-miR-676	-3.29109	0.844622	0.00248048	No, but high annotation confidence
mmu-miR-181d	-3.21633	0	0.00064655	Yes
mmu-miR-3105-5p	-3.05396	1.65299	0.0231725	No, but high annotation confidence
mmu-miR-374	-3.00234	0.87335	0.00769285	Yes, activates Wnt signaling
mmu-miR-342-5p	-2.82937	1.08799	0.0117668	Yes
mmu-miR-3105-3p	-2.80034	0	0.000739993	No, but high annotation confidence
mmu-miR-30c-2-star	-2.79674	0.87335	0.00765679	Yes
mmu-miR-30e	-2.75572	0	0.000125341	Yes
mmu-miR-130b-star	-2.6909	1.27744	0.0153159	Yes
mmu-miR-29c	-2.53999	0.844622	0.00272953	Yes
mmu-miR-467a	-2.52811	3.7983	0.0473174	Yes
mmu-miR-139-5p	-2.40183	1.4998	0.0164821	Yes
mmu-miR-148a	-2.30433	0.87335	0.00703237	Yes
mmu-miR-324-3p	-2.23358	0.87335	0.00563182	Yes
mmu-miR-30a-star	-2.22629	1.65299	0.0189066	Yes
mmu-miR-30e-star	-2.22045	0.844622	0.00254659	Yes
mmu-miR-342-3p	-2.18569	0	0.000785805	Yes
mmu-miR-187	-2.14584	1.65299	0.0196048	Yes
mmu-miR-574-3p	-2.14461	0.87335	0.00838685	Yes, activates Wnt signaling
mmu-miR-99a	-2.11156	0.844622	0.00139922	Yes, mir-10 family
mmu-miR-10a	-2.08292	0.844622	0.00265948	Yes, mir-10 family
mmu-miR-29b	-2.07621	1.08799	0.0104177	Yes
mmu-miR-96	-2.06108	0.87335	0.00820082	Yes
mmu-miR-20b	-1.98043	0	0.000117002	Yes
mmu-miR-467a-star	-1.96022	1.79736	0.0217838	Yes
mmu-miR-200a	-1.94185	0	1.96E-06	Yes
mmu-miR-30b-star	-1.91417	0.844622	0.000979444	Yes
mmu-miR-872	-1.89431	1.65299	0.0140327	No, but high annotation confidence
mmu-miR-10b	-1.89363	0.844622	0.00361751	Yes, mir-10 family
mmu-miR-326	-1.87856	2.45988	0.0248188	Yes
mmu-miR-25	-1.86261	0	5.41E-05	Yes
mmu-miR-26b	-1.85977	0.844622	0.00359004	Yes
mmu-miR-141	-1.83573	0.844622	0.0023491	Yes
mmu-miR-152	-1.81841	2.45988	0.021641	Yes
mmu-miR-30a	-1.81487	0	1.14E-05	Yes

Supplementary Table 2. List of microRNAs in microarray upregulated or downregulated, as noted, in G4 *mTR*$^{-/-}$ intestinal crypt epithelia with a minimum change of 1.8-fold and maximum q-value of 5%. The same crypt RNA samples used for the mRNA microarray were analyzed using Affymetrix GeneChip microRNA 3.0 Array chips. Since the microarray does not reflect the most recent changes in microRNA validity/annotation, each microRNA was verified with miRBase,org, as indicated on the last column ("misannotation" indicates a non-existent microRNA, "no" indicates that predicted targets of the microRNA has not yet been experimentally validated).

Gene	Validated in Experimental Model (Mouse/Human)
Axin2	Human
N-Myc	Human, Mouse
Myc	Human
Ctnnb1	Human, Mouse
Wnt1	Human, Mouse
Wnt3	Human, Rat
Lrp6	Human
Lef1	Human, Mouse
Cd44	Human, Mouse
Sirt1	Human, Mouse
Dll1	Human
Tcf7	Human
Lgr5	Human

Supplementary Table 3. Experimentally validated miR34a targets genes in the Wnt pathway. An initial literature search[9] was performed to find miR34a-targeted genes in the Wnt pathway, and the search was subsequently expanded to verify experimental evidence of suppression by miR34a in human or murine models[10, 11, 12, 13, 14, 15, 16, 17, 18, 19, 20, 21].

Mouse:	Forward	Reverse
β-Actin	GACATGGAGAAGATCTGGCA	GGTCTCAAACATGATCTGGGT
Lgr5	CTTCACTCGGTGCAGTGCT	CAGCCAGCTACCAAATAGGT
Axin2	CCTGGCTCCAGAAGATCACAA	AGCCTTCAGCATCCTCCTGT
TR	TCATTAGCTGTGGGTTCTGGT	TGGAGCTCCTGCGCTGACGTT
p21	TCTTCTGCTGTGGGTCAGGAG	GAGGGCTAAGGCCGAAGATG
Pot1a	GGCATTAGGGTTTTGCCAGATA	ATAGTGCAGAGCTGTCCCCATT
Terf2	CAGGTGAAGACAGGTCATCCAG	CCCCAGTTTCCTTCCCCGTAT
Noxa	ACTGTGGTTCTGGCGCAGAT	TGAGCACACTCGTCCTTCAAGT
mir34a	GGCAGTGTCTTAGCTGGTTGT	
Sfrp3 (Frzb)	GAAGGATCGGCTTGGTAAGAAA	GTGGGTGTGGCCTTTTACATTT
Wnt4	GAGGAGTGCCAATACCAGTTCC	AGCCACACTTCTCCAGTTCTCC

Human:	Forward	Reverse
β-Actin	AGAGCTACGAGCTGCCTGAC	AGCACTGTGTTGGCGTACAG
Lgr5	GAGTTACGTCTTGCGGGAAAC	TGGGTACGTGTCTTAGCTGATTA
Wnt3	AGGGCACCTCCACCATTTG	GACACTAACACGCCGAAGTCA
Terf2	GTACGGGGACTTCAGACAGAT	CGCGACAGACACTGCATAAC
Pot1	ATGGCACCAGGACACCATTTC	TGTAGCCGATGGATGTGACTTA
p21	CGATGGAACTTCGACTTTGTCA	GCACAAGGGTACAAGACAGTG
Noxa	ACCAAGCCGGATTTGCGATT	ACTTGCACTTGTTCCTCGTGG
Puma	GACCTCAACGCACAGTACGAG	AGGAGTCCCATGATGAGATTGT
Axin2	AAGGGCCAGGTCACCAAAC	CCCCCAACCCATCTTCGT
Ctnnb1	CCCACTAATGTCCAGCGTTT	AATCCACTGGTGAACCAAGC

Supplementary Table 4. qRT-PCR primer sequences for mouse and human genes.

Supplementary References

1. de Lau W*, et al.* Lgr5 homologues associate with Wnt receptors and mediate R-spondin signalling. *Nature* **476**, 293-297 (2011).

2. van der Flier LG*, et al.* Transcription factor achaete scute-like 2 controls intestinal stem cell fate. *Cell* **136**, 903-912 (2009).

3. Munoz J*, et al.* The Lgr5 intestinal stem cell signature: robust expression of proposed quiescent '+4' cell markers. *EMBO J* **31**, 3079-3091 (2012).

4. Takeda N, Jain R, LeBoeuf MR, Wang Q, Lu MM, Epstein JA. Interconversion between intestinal stem cell populations in distinct niches. *Science* **334**, 1420-1424 (2011).

5. Maria Cambuli F, Rezza A, Nadjar J, Plateroti M. Brief report: Musashi1-eGFP mice, a new tool for differential isolation of the intestinal stem cell populations. *Stem Cells* **31**, 2273-2278 (2013).

6. Zhao J*, et al.* R-spondin1, a novel intestinotrophic mitogen, ameliorates experimental colitis in mice. *Gastroenterology* **132**, 1331-1343 (2007).

7. Merlos-Suarez A*, et al.* The intestinal stem cell signature identifies colorectal cancer stem cells and predicts disease relapse. *Cell Stem Cell* **8**, 511-524 (2011).

8. Crabbe L, Verdun RE, Haggblom CI, Karlseder J. Defective telomere lagging strand synthesis in cells lacking WRN helicase activity. *Science* **306**, 1951-1953 (2004).

9. Rokavec M, Li H, Jiang L, Hermeking H. The p53/miR-34 axis in development and disease. *J Mol Cell Biol* **6**, 214-230 (2014).

10. Kim NH*, et al.* p53 regulates nuclear GSK-3 levels through miR-34-mediated Axin2 suppression in colorectal cancer cells. *Cell Cycle* **12**, 1578-1587 (2013).

11. Wei JS*, et al.* The MYCN oncogene is a direct target of miR-34a. *Oncogene* **27**, 5204-5213 (2008).

12. Yamamura S*, et al.* MicroRNA-34a modulates c-Myc transcriptional complexes to suppress malignancy in human prostate cancer cells. *PLoS One* **7**, e29722 (2012).

13. Yamamura S*, et al.* MicroRNA-34a suppresses malignant transformation by targeting c-Myc transcriptional complexes in human renal cell carcinoma. *Carcinogenesis* **33**, 294-300 (2012).

14. Kim NH*, et al.* p53 and microRNA-34 are suppressors of canonical Wnt signaling. *Sci Signal* **4**, ra71 (2011).

15. Hashimi ST, Fulcher JA, Chang MH, Gov L, Wang S, Lee B. MicroRNA profiling identifies miR-34a and miR-21 and their target genes JAG1 and WNT1 in the coordinate regulation of dendritic cell differentiation. *Blood* **114**, 404-414 (2009).

16. Liu FJ*, et al.* microRNAs Involved in Regulating Spontaneous Recovery in Embolic Stroke Model. *PLoS One* **8**, e66393 (2013).

17. Liu C*, et al.* The microRNA miR-34a inhibits prostate cancer stem cells and metastasis by directly repressing CD44. *Nat Med* **17**, 211-215 (2011).

18. Yamakuchi M, Ferlito M, Lowenstein CJ. miR-34a repression of SIRT1 regulates apoptosis. *Proc Natl Acad Sci U S A* **105**, 13421-13426 (2008).

19. Xiong H*, et al.* Activation of miR-34a/SIRT1/p53 signaling contributes to cochlear hair cell apoptosis: implications for age-related hearing loss. *Neurobiol Aging* **36**, 1692-1701 (2015).

20. de Antonellis P*, et al.* MiR-34a targeting of Notch ligand delta-like 1 impairs CD15+/CD133+ tumor-propagating cells and supports neural differentiation in medulloblastoma. *PLoS One* **6**, e24584 (2011).

21. Chen WY*, et al.* MicroRNA-34a regulates WNT/TCF7 signaling and inhibits bone metastasis in Ras-activated prostate cancer. *Oncotarget* **6**, 441-457 (2015).
