# Supplementary material for: Mutual reinforcement between telomere capping and canonical Wnt signalling in the intestinal stem cell niche
Source: Nat Commun. 2017 Mar 17;8:14766. doi: 10.1038/ncomms14766 (PMC5357864; doi:10.1038/ncomms14766)
Supplement: Peer Review File [file ncomms14766-s2.pdf]

Editorial Note: this manuscript has been previously reviewed at another journal that is not operating a transparent peer review scheme. This document only contains reviewer comments and rebuttal letters for versions considered at Nature Communications.

REVIEWERS' COMMENTS:

Reviewer #1 (Remarks to the Author):

The authors have addressed the remaining concerns in a thoroughly argued rebuttal, and with text changes and new data strengthening the manuscript. My only minor suggestion is that the discussion reference to the danazol study could be omitted as it seems unrelated, and mechanisms/implications of the altered measures of telomere length in that study remain unclear.

We thank the reviewer for their positive assessment of our previously revised manuscript. The reviewer had one remaining concern: "My only minor suggestion is that the discussion reference to the danazol study should be omitted as it seems unrelated, and mechanisms/implications of the altered measures of telomere length in that study remain unclear."

To respond to this concern, we have removed the relevant sentence.
